# Cyclometalated iridium(III) chelates—a new exceptional class of the electrochemiluminescent luminophores

**DOI:** 10.1007/s00216-016-9615-8

**Published:** 2016-06-02

**Authors:** Andrzej Kapturkiewicz

**Affiliations:** Institute of Chemistry, Faculty of Sciences, Siedlce University of Natural Sciences and Humanities, 3 Maja 54, 08-110 Siedlce, Poland

**Keywords:** Cyclometalated iridium(III) chelates, Electrogenerated chemiluminescence (ECL), Electroanalytical methods, Fluorescence/luminescence

## Abstract

Recent development of the phosphorescent cyclometalated iridium(III) chelates has enabled, due to their advantageous electrochemical and photo-physical properties, important breakthroughs in many photonic applications. This particular class of 5d^6^ ion complexes has attracted increasing interest because of their potential application in electroluminescence devices with a nearly 100 % internal quantum efficiency for the conversion of electric energy to photons. Similar to electroluminescence, the cyclometalated iridium(III) chelates have been successfully applied in the electricity-to-light conversion by means of the electrochemiluminescence (ECL) processes. The already reported ECL systems utilizing the title compounds exhibit extremely large ECL efficiencies that allow one to envisage many potential application for them, especially in further development of ECL-based analytical techniques. This review, based on recently published papers, focuses on the ECL properties of this very exciting class of organometallic luminophores. The reported work, describing results from fundamental as well as application-oriented investigations, will be surveyed and briefly discussed.

**Graphical abstract:**
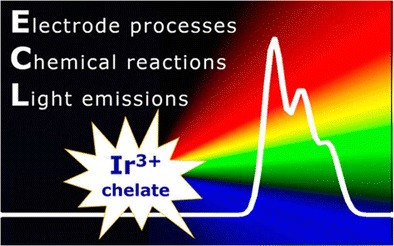
Depending on the chemical nature of the cyclometalated irdium(III) chelate different colours of the emitted light can be produced during electrochemical excitation.

Depending on the chemical nature of the cyclometalated irdium(III) chelate different colours of the emitted light can be produced during electrochemical excitation.

## Introduction

Electrogenerated chemiluminescence (also called electrochemiluminescence and abbreviated ECL) is defined as the emission of light resulting from generation of the electronically excited states populated in the electron transfer process between the species generated at electrodes. The phenomenon, known for at least 50 y from the pioneering work of Hercules [1], Visco and Chandross [2], and Santhanam and Bard [3], has now become a very powerful and widely used analytical technique. After the first observation of ECL emission, this exciting technique has been extensively investigated, including the discovery of new ECL luminophores, explanation of the mechanisms associated with light generation, and, last but not least, the development of practical applications.

Until now ECL studies were reported (according to date form Web of Science Core Collection) in ca. 8000 journal articles with overall number of papers increasing exponentially over recent years. Most of the articles are related to different analytical, bioanalytical, and clinical analysis applications. A considerable number of reviews on a wide variety of ECL subjects have been also published. Appropriate references can be found in the first comprehensive ECL monograph edited by Bard [4]. The Bard monograph summarizes ECL achievements finalized before 2003, whereas further ECL development is summarized in the review papers published thereafter [5–21]. The number of recently published ECL reviews clearly points to considerable progress in the field, especially considering analytical applications of ECL technique. Already developed practical applications include ECL as a very powerful analytical method used in areas such as immunoassay, food and water testing, and bioactive species detection. ECL systems have also been successfully exploited as a detector in flow injection analysis, high pressure liquid chromatography, capillary electrophoresis, and micro total analysis. For more detail concerning analytical application of ECL technique, the reader is referred to compressive ECL monograph and reviews mentioned above.

ECL analytical applications are mostly based on the famous ECL luminophore *tris*(2,2′-bipyridine)ruthenium(II) cation – Ru(bpy)_3_^2+^ – involved in the first ECL system based on the luminescent transition metal complex discovered at the beginning of the 1970s [22]. In this ECL system, light emission arises from the excited metal-to-ligand charge transfer (MLCT) triplet state ^3*^Ru(bpy)_3_^2+^ populated in the electron transfer annihilation between the oxidized Ru(bpy)_3_^3+^ and the reduced Ru(bpy)_3_^1+^ species generated in acetonitrile solutions [23] by means of electrochemical oxidation and reduction.

1a $$ \mathrm{R}\mathrm{u}{\left(\mathrm{b}\mathrm{p}\mathrm{y}\right)}_3^{2+}+{e}^{-}\rightleftharpoons \mathrm{R}\mathrm{u}{\left(\mathrm{b}\mathrm{p}\mathrm{y}\right)}_3^{1+} $$

1b $$ \mathrm{R}\mathrm{u}{\left(\mathrm{b}\mathrm{p}\mathrm{y}\right)}_3^{2+}-{e}^{-}\rightleftharpoons \mathrm{R}\mathrm{u}{\left(\mathrm{b}\mathrm{p}\mathrm{y}\right)}_3^{3+} $$

1c $$ \mathrm{R}\mathrm{u}{\left(\mathrm{b}\mathrm{p}\mathrm{y}\right)}_3^{3+}+\mathrm{R}\mathrm{u}{\left(\mathrm{b}\mathrm{p}\mathrm{y}\right)}_3^{1+}\to {}^{3\ast}\mathrm{R}\mathrm{u}{\left(\mathrm{b}\mathrm{p}\mathrm{y}\right)}_3^{2+}+\mathrm{R}\mathrm{u}{\left(\mathrm{b}\mathrm{p}\mathrm{y}\right)}_3^{2+} $$

1d $$ \mathrm{R}\mathrm{u}{\left(\mathrm{b}\mathrm{p}\mathrm{y}\right)}_3^{3+}+\mathrm{R}\mathrm{u}{\left(\mathrm{b}\mathrm{p}\mathrm{y}\right)}_3^{1+}\to \mathrm{R}\mathrm{u}{\left(\mathrm{b}\mathrm{p}\mathrm{y}\right)}_3^{2+}+\mathrm{R}\mathrm{u}{\left(\mathrm{b}\mathrm{p}\mathrm{y}\right)}_3^{2+} $$

The overall actinometrically determined ECL efficiency (*ϕ*_ecl_ expressed in emitted photons produced per annihilation event) has been found to be close to the luminescence quantum yields *ϕ*_em_ of the excited ^3*^Ru(bpy)_3_^2+^ state. The *ϕ*_ecl_ value (0.05 at room temperature) found for the Ru(bpy)_3_^3+^/Ru(bpy)_3_^1+^ annihilation is commonly used as the efficiency standard in any other ECL investigations. The found ECL efficiency approaches the intrinsic luminescence efficiency of the populated emitter, allowing to conclude that (i) the formation efficiency of the excited ^3*^Ru(bpy)_3_^2+^ species (Equation 1c) is close unity, and (ii) the thermodynamically favored direct formation of the ground-state products (Equation 1d) is kinetically inhibited in accordance with the Marcus model of electron transfer excitation [24].

Many other studies concerning Ru(bpy)_3_^2+^ ion have followed, with the first report of ECL in an aqueous solution involving Ru(bpy)_3_^2+^ cation and the oxalate C_2_O_4_^2−^ anion [25]. Subsequently, other species, peroxydisulfate ion S_2_O_8_^2−^ [26] and tri-*n*-propylamine (*n*-C_3_H_7_)_3_N – TPrA [27] were shown to act as active ECL co-reactants. The latter of the above-mentioned ECL system, based on parallel electrochemical oxidation of Ru(bpy)_3_^2+^ and TPrA species, became a base of many ECL-based analytical techniques. At the present time, nearly all commercially available ECL analytical instruments are based on appropriate modifications of this technology.

The Ru(bpy)_3_^2+^/TPrA combination, despite its versatility, presents some disadvantages, arising mainly from relatively low value of the emission quantum yields (only a few percent) characterizing the excited ^3*^Ru(bpy)_2_^3+^ state. The second limiting factor is the efficiency of ^3*^Ru(bpy)_2_^3+^ population in the electron transfer process involving the intrinsic substrates of electrochemical excitation, namely Ru(bpy)_3_^3+^ and TPrA^•^ radical (CH_3_CH_2_CH_2_)_2_NC^•^HCH_2_CH_3_, the latter produced by means of proton abstraction from the electrochemically generated (CH_3_CH_2_CH_2_)_3_N^+^ radical cation. Taking into account the presence of the restrictions pointed out above, one can conclude that any another combination of an ECL co-reactant and/or an ECL luminophore may still be better from the sensitivity and the detection limit points of view, of course if the above mentioned limiting factors could be overcome. Work already reported in this direction clearly indicates that this is not the only promising option. For example, tertiary amines, such as tri-*iso*-butylamine − TBA [28, 29], or 2-(dibutylamino)ethanol − DBAE [30], have been found to give much more intense ECL signal than TPrA in commonly used Ru(bpy)_3_^2+^/amine systems. In a similar manner, new ECL luminophores, usually transition metal chelates, sometimes much better emissive than Ru(bpy)_3_^2+^, have also been reported. Among many investigated metal/ligand(s) combinations, particular attention has been paid to the cyclometalated iridium(III) chelates, an outstanding class of the extremely efficient organometallic emitters.

**Fig. 1:**
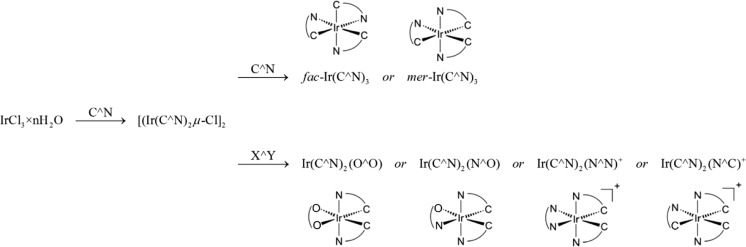
Synthetic routes and molecular structures of the homoleptic Ir(C^N)_3_ and heteroleptic Ir(C^N)_2_(X^Y) complexes

This mini-review is specifically devoted to ECL studies of the cyclometalated iridium(III) chelates, selected because of their potential significance in further ECL progress. Specific character of this review precludes detailed description of ECL phenomenon and the techniques applied in ECL investigations. The reader not familiar with the subjects is referred to the above-mentioned ECL monograph or reviews and references cited therein. Similarly, the title iridium(III) complexes cannot be exhaustively reviewed in this work. After the first papers from Demas and coworkers [31–33] and Watt et al. [34–36] reporting synthesis and photo-physical properties of the cyclometalated iridium(III) chelates, this class of organometallic luminophores has been extensively studied with exploding interest over the last few years. The recent progress in chemistry of the cyclometalated iridium(III) complexes, their synthesis, properties, and application have been presented in many review articles [37–46] undoubtedly emphasizing unique properties of these chelates. Molecular structures and synthetic routes for different types of the cyclometalated iridium(III) complexes used in the reviewed ECL studies are schematically depicted in Fig. 1.

## ECL properties of the homoleptic iridium(III) chelates

**Fig. 2:**
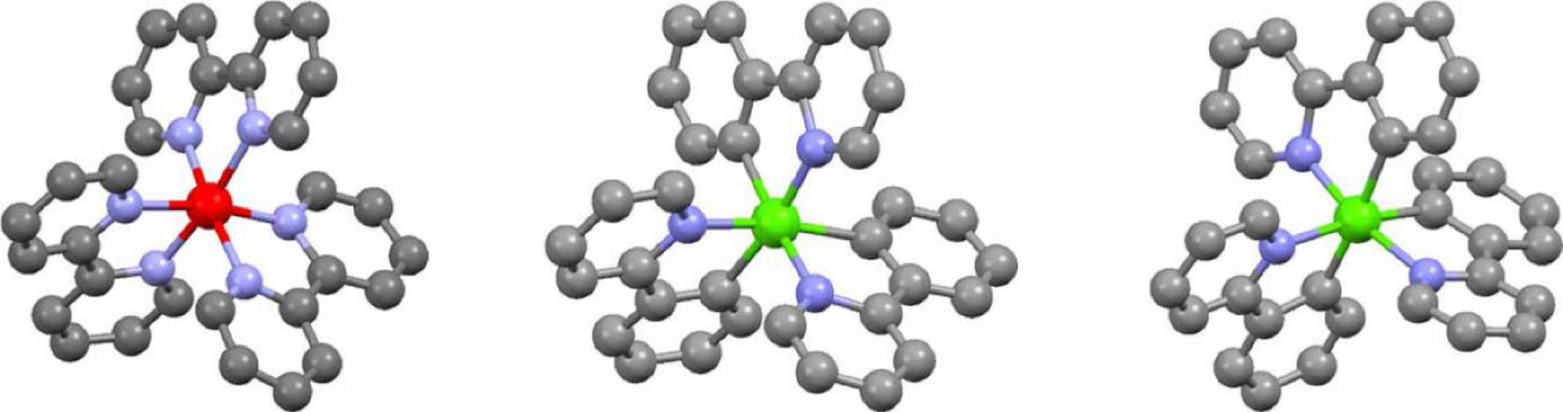
Structures of Ru(bpy)_3_^2+^ (left), *fac*-Ir(ppy)_3_ (middle), and *mer*-Ir(ppy)_3_ (*right*) complexes. Atoms color: black – carbon, blue – nitrogen, red – ruthenium, and green – iridium. Hydrogen atoms are omitted for clarity

The general structure of the homoleptic cyclometalated iridium(III) complexes includes one iridium(III) core ion surrounded by three equal bidentate monoanionic ligands. Usually, the atoms in the ligand, which are bonded to the iridium(III) central ion, are N and C (with a formal negative charge) with the coordinative disposition involving the formation of a five- or six-membered metallacycle. These C^N ligands and the resulting complexes are named cyclometalating ligands and cyclometalated complexes, respectively. Most of the cyclometalating ligands have one neutral coordinating part and one anionic part. The Ir–C bond between the iridium(III) and carbon atom of C^N ligands is usually strong enough to be comparable to covalent bonds that leads to a multiply bonded and compact structure with extensive electronic interactions between the d-orbital of iridium(III) ion and π-orbital of the ligands. These interactions, together with a large spin-orbit coupling effect caused by the presence of heavy metal ion, are responsible for the well pronounced room-temperature phosphorescence characteristic for the cyclometalated iridium(III) chelates. Octahedral coordination arrangement of an iridium(III) complex allows for the presence of two geometric isomers with facial (*fac*-) or meridional (*mer*-) conformation (cf. Fig. 2) with somewhat different electrochemical, spectroscopic, and photo-physical properties [47]. Isolated samples of *mer*-Ir(C^N)_3_ chelates can be thermally and photo-chemically converted to facial, thermodynamically more stable forms, usually a distinctly superior emissive compared with their meridional counterparts.

**Fig. 3:**
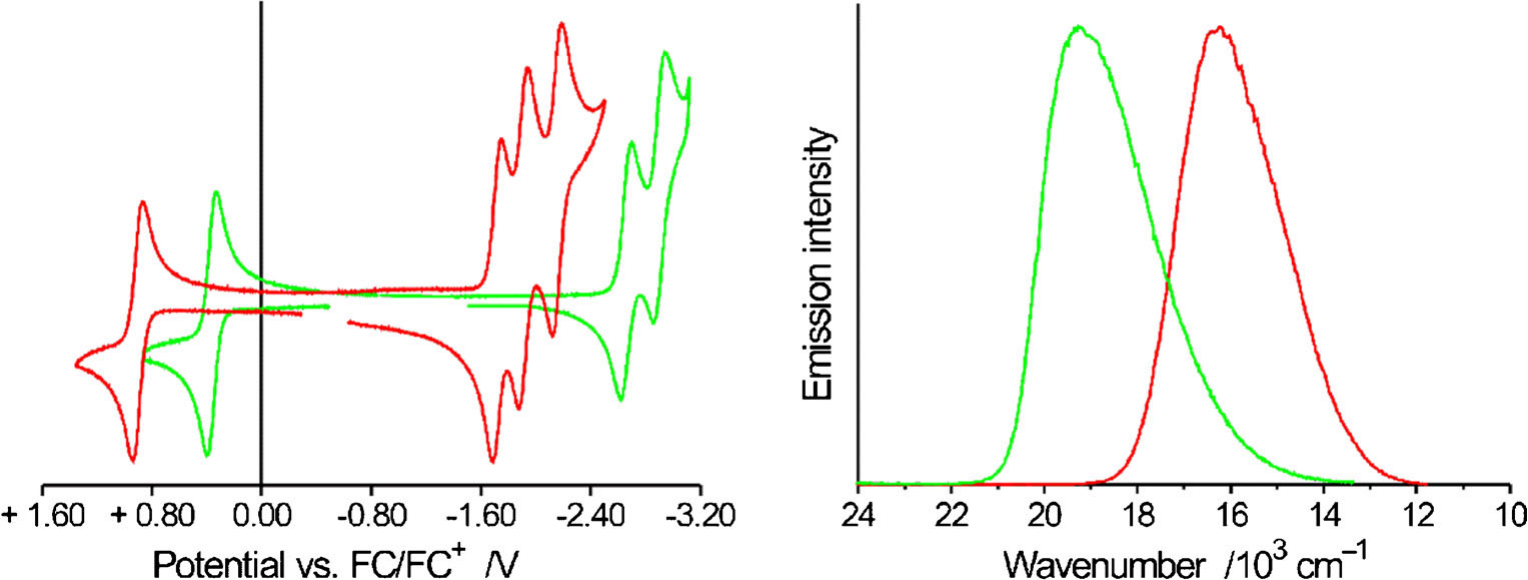
Cyclic voltammograms (left) and ECL emission spectra (right) of Ru(bpy)_3_^2+^ (red lines) and Ir(ppy)_3_ (green lines) chelates recorded in the Author laboratory. Data for 1 mM of Ru(bpy)_3_^2+^ in 0.1 M (*n*-C_4_H_9_)_4_NPF_6_/acetonitrile solutions and 1 mM of Ir(pp)_3_ in 0.1 M (*n*-C_4_H_9_)_4_NPF_6_/acetonitrile-dioxane-1:1 solutions, respectively. For both complexes, reversible one-electron oxidation corresponds to the removal of a metal t_2g_ orbital whereas a series of reversible one-electron reductions with the added electrons localized on individual ligand π* orbitals. A third reduction wave, corresponding to Ir(ppy)_3_^3−^ formation could not be observed under the experimental conditions because of the redox potential value being more negative than the solvent cathodic limit. Adapted from ref [10]

Among many different C^N ligands that could be attached to iridium(III) core, the most popular is 2-phenylpyridine (ppyH) forming facial isomer of Ir(ppy)_3_ chelate, particularly interesting from the ECL point of view because the neutral Ir(ppy)_3_ molecule is formally isoelectronic with Ru(bpy)_3_^2+^ cation. The isoelectronic character of these two species allowed one to expect observation of the ECL emission from Ir(ppy)_3_ chelate as it has been presented in the preliminary reports published by Fuyuki et al. [48], Wightman et al. [49], and Bruce and Richter [50] just at the beginning of the 21st century. More detailed investigations, presented in 2003 by Kapturkiewicz and Angulo [51], have quantitatively confirmed the expected similarities in mechanism of the electrochemical generation of the excited triplet ^3*^Ru(bpy)_3_^2+^ and ^3*^Ir(ppy)_3_ states. Similar to the Ru(bpy)_3_^2+^ ion, the Ir(ppy)_3_ complex can be reversibly oxidized and reduced (cf. Fig. 3) to the stable Ir(ppy)_3_^+^ cation and Ir(ppy)_3_^−^ anion

2a $$ \mathrm{I}\mathrm{r}{\left(\mathrm{p}\mathrm{p}\mathrm{y}\right)}_3+{e}^{-}\rightleftharpoons \mathrm{I}\mathrm{r}{{\left(\mathrm{p}\mathrm{p}\mathrm{y}\right)}_3}^{-} $$

2b $$ \mathrm{I}\mathrm{r}{\left(\mathrm{p}\mathrm{p}\mathrm{y}\right)}_3-{e}^{-}\rightleftharpoons \mathrm{I}\mathrm{r}{{\left(\mathrm{p}\mathrm{p}\mathrm{y}\right)}_3}^{+} $$

Electron transfer annihilation of the electrochemically produced Ir(ppy)_3_^+^ and Ir(ppy)_3_^−^ species leads then to the generation of the excited ^3*^Ir(ppy)_3_ state according to

3a $$ \mathrm{I}\mathrm{r}\kern0.28em {{\left(\mathrm{p}\mathrm{p}\mathrm{y}\right)}_3}^{+}+\mathrm{I}\mathrm{r}{{\left(\mathrm{p}\mathrm{p}\mathrm{y}\right)}_3}^{-}\to {}^{3\ast}\mathrm{I}\mathrm{r}{\left(\mathrm{p}\mathrm{p}\mathrm{y}\right)}_3+\mathrm{I}\mathrm{r}{\left(\mathrm{p}\mathrm{p}\mathrm{y}\right)}_3 $$

3b $$ \mathrm{I}\mathrm{r}{{\left(\mathrm{p}\mathrm{p}\mathrm{y}\right)}_3}^{+}+\mathrm{I}\mathrm{r}{{\left(\mathrm{p}\mathrm{p}\mathrm{y}\right)}_3}^{-}\to \mathrm{I}\mathrm{r}{\left(\mathrm{p}\mathrm{p}\mathrm{y}\right)}_3+\mathrm{I}\mathrm{r}{\left(\mathrm{p}\mathrm{p}\mathrm{y}\right)}_3 $$

The ECL spectra (cf. Fig. 3), recorded using the triple-potential-step technique, were found to be identical with the photoluminescence spectra indicating formation of the same metal-to-ligand (MLCT) excited state in both excitation processes. ECL emission efficiency for the single Ir(ppy)_3_^−^/Ir(ppy)_3_^+^ system as high as 0.16 (ca. three times larger than 0.05 characteristic for Ru(bpy)_3_^3+^/Ru(bpy)_3_^+^ pair) has been found during experiments performed in acetonitrile/dioxane solutions containing 0.1 M (C_4_H_9_)_4_NPF_6_ as the supporting electrolyte. It has also been found that the electron transfer between the electrochemically generated radical anions of aromatic nitriles and ketones A^−^ and Ir(ppy)_3_^+^ cation allows the direct population of the excited strongly emissive ^3*^Ir(ppy)_3_ species with still higher yields [51].

4a $$ \mathrm{I}\mathrm{r}{{\left(\mathrm{p}\mathrm{p}\mathrm{y}\right)}_3}^{+}+{\mathrm{A}}^{-}\to {}^{3\ast}\mathrm{I}\mathrm{r}{\left(\mathrm{p}\mathrm{p}\mathrm{y}\right)}_3+\mathrm{A} $$

4b $$ \mathrm{I}\mathrm{r}{{\left(\mathrm{p}\mathrm{p}\mathrm{y}\right)}_3}^{+}+{\mathrm{A}}^{-}\to \mathrm{I}\mathrm{r}{\left(\mathrm{p}\mathrm{p}\mathrm{y}\right)}_3+\mathrm{A} $$

The *ϕ*_ecl_ value of 0.67 (close to the excited ^3*^Ir(ppy)_3_ luminescence quantum yield *ϕ*_em_ of 0.75) has been found for the Ir(ppy)_3_^+^/2-cyanofluorene^−^ system. The *ϕ*_ecl_ value of 0.67 is most probably the highest ECL efficiency found until now. According to the above-presented reaction scheme, the ECL efficiencies *ϕ*_ecl_ are directly related to the yield of the excited state generation *ϕ*_es_ and to the emission quantum yield *ϕ*_em_ of a given emitter.

5). $$ {\phi}_{\mathrm{ecl}}={\phi}_{\mathrm{es}}{\phi}_{\mathrm{em}} $$

**Fig. 4:**
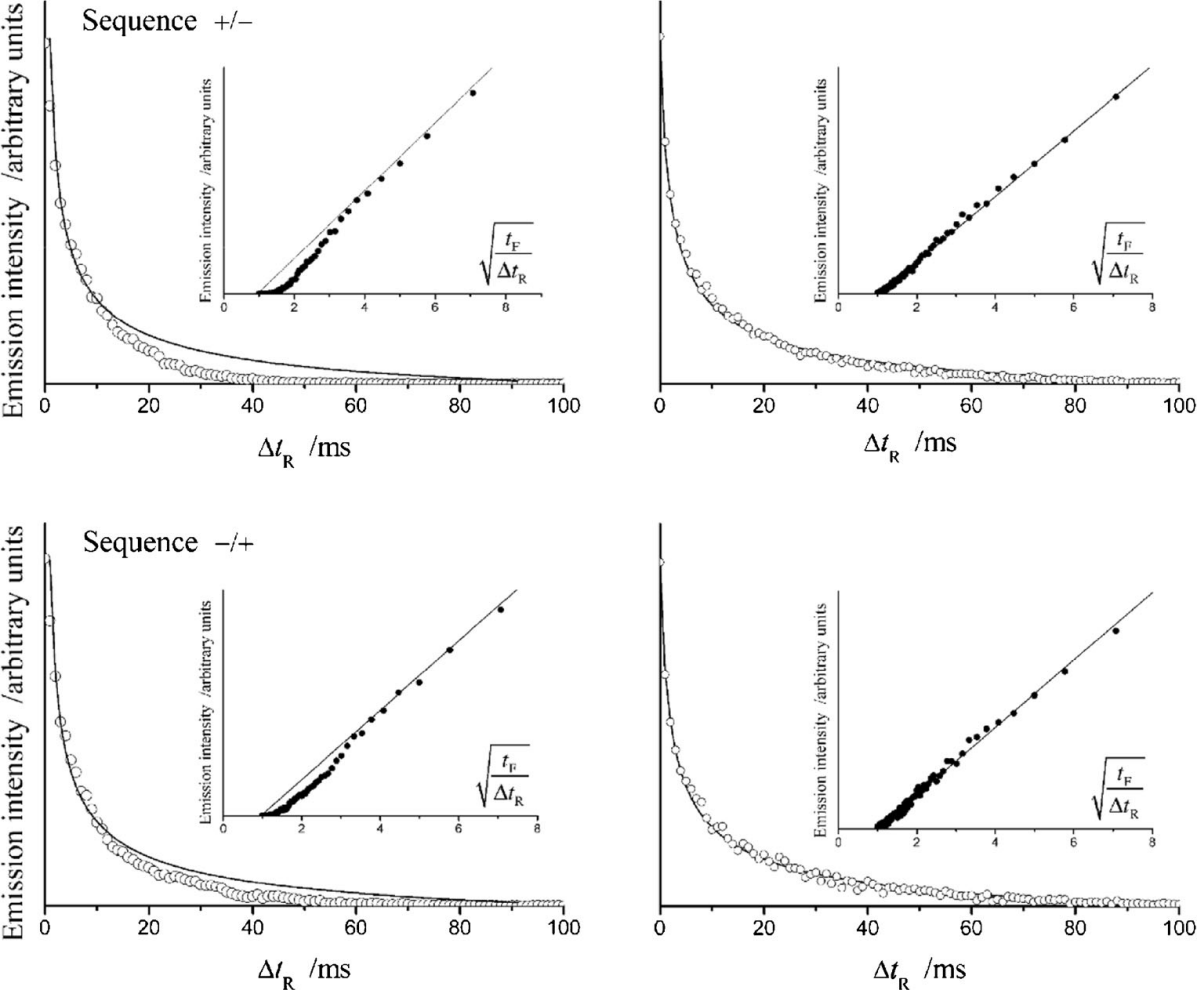
ECL decay curves and plots of intensities *I*(Δ*t*_R_) versus (*t*_F_/Δ*t*_R_)^1/2^ recorded for the single Ir(ppy)_3_^+^/Ir(ppy)_3_^−^ and the mixed Ir(ppy)_3_^+^/benzophenone^−^ system in acetonitrile-dioxane-1:1 solutions containing 0.1 M (*n*-C_4_H_9_)_4_NPF_6_ as supporting electrolyte. Concentration of Ir(ppy)_3_ or benzophenone reactants equal to 1 mM. Sequences −/+ and +/− denote the order of the reactant generations (first reductant or first oxidant, respectively) in a triple-potential-step experiments. Forward and reverse pulse duration times (*t*_F_ and *t*_R_, correspondingly) were 100 ms for both cases. Δ*t*_R_ denotes the time delay from the start of the second reverse potential step. For the mixed Ir(ppy)_3_^+^/benzophenone^−^ and other investigated Ir(ppy)_3_^+^/A^−^ systems *I*(Δ*t*_R_) transients have been linearized according to so-called Feldberg plot *I*(Δ*t*_R_) = *a*(*t*_F_/Δ*t*_R_)^1/2^ − *b* [52] with the slope-to intercept ratios found to be close to the theoretical value of 0.959, pointing to direct formation of the emitting ^3*^Ir(ppy)_3_ in the annihilation of Ir(ppy)_3_^+^ and A^−^ ions. Intrinsic deviation from linearity in the Feldberg plots found for the single Ir(ppy)_3_^+^/Ir(ppy)_3_^−^ system suggesting the presence of some additional parasitic processes. Adapted from ref [51]

Thus, in the case of some Ir(ppy)_3_^+^/A^−^ systems, the excited ^3*^Ir(ppy)_3_ state can be populated with nearly 100 % yields, similarly as found for the Ru(bpy)_3_^3+^/Ru(bpy)_3_^+^ system. For the Ir(ppy)_3_^+^/Ir(ppy)_3_^−^ pair, however, a distinctly smaller *ϕ*_es_ value has been observed despite similar annihilation exergonicities. Analysis of the ECL transients (cf. Fig. 4) allowed one to determine presence of any additional parasitic processes occurring during the electrochemical excitation within the single Ir(ppy)_3_^+^/Ir(ppy)_3_^−^ system. It is important to note that similar effects are not observed for the mixed Ir(ppy)_3_^+^/A^−^ systems that may explain the observation of larger *ϕ*_ecl_ and *ϕ*_es_ values.

The energy released during the annihilation of Ir(ppy)_3_^+^ and Ir(ppy)_3_^−^ ions is sufficiently negative (−3.01 eV as calculated from the difference in *E*_ox_ and *E*_red_ potentials) to directly populate (Equation 3a) the excited ^3*^Ir(ppy)_3_ state with energy of 2.50 eV. The free energy for the ground-state product formation (Equation 3b) is correspondingly so negative that this pathway, deeply lying in the Marcus inverted region, is inhibited. Consequently, the energetically accessible excited-state formation process is dominant, leading to extremely efficient ECL excitation. The sufficiently low energy of the excited ^3*^Ir(ppy)_3_ state also allows for the experimental observation of the ECL phenomenon in so-called mixed systems (e.g., in the reactions between Ir(ppy)_3_^+^ and strong reductants A^−^). For this ECL excitation route, it has been found that partitioning between the simultaneously occurring processes leading to the ground-state (Equation 4b) and excited-state (Equation 4a) products depends strongly on the energy released during electron transfer annihilation. Over a narrow free energy range, *ϕ*_ecl_ rapidly increases in a way consistent with the Marcus model predictions [24], to quasi-plateau value for sufficiently exergonic systems, as it was previously found for the ECL systems involving Mo_6_Cl_14_^2−^ clusters ion [53], Ru(bpy)_3_^2+^ ion [54], or other Ru(α-diimine)_3_^2+^ chelates [55]. Of note, the electron annihilation energetics do not seem to be the only factor governing the observed efficiencies of the electrochemical excitation. Both the threshold energy and the limiting *ϕ*_ecl_ value for the given ECL luminophore have been found to depend on the chemical nature of organic auxiliary reactants. This indicates that the partitioning between the simultaneously occurring processes leading to the ground- and excited-state products may be much more complex than predicted by the Marcus model. An appropriate explanation of the observed inconsistencies remains an open question requiring further work that could possibly produce a decisive answer.

**Fig. 5:**
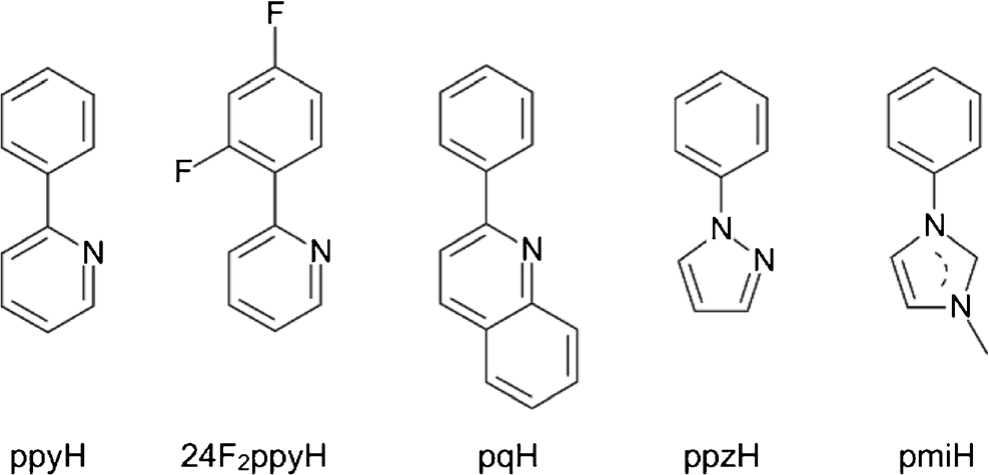
Structures of the protonated forms of C^N and C^C ligands used in ECL studies of the cyclometalated homoleptic Ir(C^N)_3_ and Ir(C^C)_3_ chelates

Considering prospective ECL behavior of other homoleptic Ir(C^N)_3_ chelates, one can expect that analogues of Ir(ppy)_3_ (e.g., with the substituted 2-phenylpyridines or any congeneric heteroaromatic compounds as the cyclometalating C^N ligands) should follow the same electrochemical excitation mechanism. Somewhat surprisingly, however, ECL properties of Ir(ppy)_3_ analogues remain nearly unexplored, despite different homoleptic cyclometalated iridium(III) complexes that are quite extensively described in the literature [56–61]. Most probably, harsh reaction conditions required to synthesize these complexes, rather low yields of the required products, and necessary large excess of sometimes hardly accessible C^N ligands make Ir(C^N)_3_ chelates unfavorable for investigations in the ECL-oriented laboratories. Until now, besides of Ir(ppy)_3_, only homoleptic Ir(C^N)_3_ complexes with the deprotonated forms of 2-phenylquinoline − pqH [62], 2-(2,4-difluorophenylo)pyridine − 24F_2_ppyH [63–65], and 1-phenylpyrazol – ppzH [66] as the C^N cyclometalating ligands (structures depicted in Fig. 5) have been reported as active ECL materials. Preliminary ECL studies have also been performed for the Ir(C^C)_3_ complex with 1-phenyl-3-methylimidazolin-2-ylidene – pmiH acting as the C^C cyclometalating ligand [66].

Compared with Ru(bpy)_3_^3+^/Ru(bpy)_3_^+^, ECL system electron transfer annihilation between Ir(pq)_3_^+^ and Ir(pq)_3_^−^ ions was demonstrated to be more intense (by a factor of 3.42) ECL emission in acetonitrile solutions. In a similar way, the combination of Ir(pq)_3_ and TPrA co-reactant was found to be more efficient (by a factor of 2.73–14.18) compared with the Ru(bpy)_3_^2+^/TPrA reference system [62]. Ir(24F_2_ppy)_3_, Ir(ppz)_3_, and Ir(pmi)_3_ chelates have been tested as a blue-emissive component for multiplexed, multicolored ECL generated with the use of TPrA co-reactant [63–66]. The solution phase and solid-state electrochemistry and ECL studies of a *fac*-Ir(ppy)_3_-cored dendrimer have been recently reported by Hogan et al. [67]. Dendritic architecture has been tested as shielding the emissive ^3*^Ir(ppy)_3_ core from any luminescence perturbation (e.g., due to oxygen quenching processes).

Light emission in the above-listed examples of Ir(C^N)_3_ chelates arises from the excited ^3*^Ir(C^N)_3_ population by means of the electron transfer between Ir(C^N)_3_^+^ cation and TPrA^•^ radical (deprotonated form of TPrA^+^ radical cation), both produced during electrochemical oxidation of Ir(C^N)_3_ and TPrA species:

6a $$ \mathrm{I}\mathrm{r}{\left(\mathrm{C}\hat{\mkern6mu} \mathrm{N}\right)}_3-{e}^{-}\rightleftarrows \mathrm{I}\mathrm{r}{{\left(\mathrm{C}\hat{\mkern6mu} \mathrm{N}\right)}_3}^{+} $$

6b $$ {\left(n\hbox{-} {\mathrm{C}}_3{\mathrm{H}}_7\right)}_3\mathrm{N}-{e}^{-}\rightleftarrows {\left(n\hbox{-} {\mathrm{C}}_3{\mathrm{H}}_7\right)}_3{\mathrm{N}}^{+}\to {\left(n\hbox{-} {\mathrm{C}}_3{\mathrm{H}}_7\right)}_2{\mathrm{N}\mathrm{C}}^{\bullet }{\mathrm{H}\mathrm{C}\mathrm{H}}_2{\mathrm{C}\mathrm{H}}_3 $$

6c $$ \mathrm{I}\mathrm{r}{{\left(\mathrm{C}\hat{\mkern6mu} \mathrm{N}\right)}_3}^{+}+{\left(n\hbox{-} {\mathrm{C}}_3{\mathrm{H}}_7\right)}_2{\mathrm{NC}}^{\bullet }{\mathrm{H}\mathrm{CH}}_2{\mathrm{C}\mathrm{H}}_3\to {}^{3\ast}\mathrm{I}\mathrm{r}{\left(\mathrm{C}\hat{\mkern6mu} \mathrm{N}\right)}_3+{\left(n\hbox{-} {\mathrm{C}}_3{\mathrm{H}}_7\right)}_2{\mathrm{NC}}^{+}{\mathrm{H}\mathrm{CH}}_2{\mathrm{C}\mathrm{H}}_3 $$

Using the analogy between ECL excitation of Ru(bpy)_3_^2+^ and Ir(C^N)_3_ luminophores one can also speculate about some additional processes that can take place as well:

6d $$ \mathrm{I}\mathrm{r}{\left(\mathrm{C}\hat{\mkern6mu} \mathrm{N}\right)}_3+{\left(n\hbox{-} {\mathrm{C}}_3{\mathrm{H}}_7\right)}_3{\mathrm{N}}^{+}\rightleftarrows \mathrm{I}\mathrm{r}{{\left(\mathrm{C}\hat{\mkern6mu} \mathrm{N}\right)}_3}^{+}+{\left(n\hbox{-} {\mathrm{C}}_3{\mathrm{H}}_7\right)}_3\mathrm{N} $$

6e $$ \mathrm{I}\mathrm{r}{\left(\mathrm{C}\hat{\mkern6mu} \mathrm{N}\right)}_3+{\left(n\hbox{-} {\mathrm{C}}_3{\mathrm{H}}_7\right)}_2{\mathrm{NC}}^{\bullet }{\mathrm{H}\mathrm{CH}}_2{\mathrm{C}\mathrm{H}}_3\rightleftarrows \mathrm{I}\mathrm{r}{{\left(\mathrm{C}\hat{\mkern6mu} \mathrm{N}\right)}_3}^{-}+{\left(n\hbox{-} {\mathrm{C}}_3{\mathrm{H}}_7\right)}_2{\mathrm{NC}}^{+}{\mathrm{H}\mathrm{CH}}_2{\mathrm{C}\mathrm{H}}_3 $$

6f $$ \mathrm{I}\mathrm{r}{{\left(\mathrm{C}\hat{\mkern6mu} \mathrm{N}\right)}_3}^{-}+{\left(n{\textstyle \hbox{-} }{\mathrm{C}}_3{\mathrm{H}}_7\right)}_3{\mathrm{N}}^{+}\to {}^{3\ast}\mathrm{I}\mathrm{r}{\left(\widehat{\mathrm{C}}\mathrm{N}\right)}_3+{\left(n{\textstyle \hbox{-} }{\mathrm{C}}_3{\mathrm{H}}_7\right)}_3\mathrm{N} $$

6g $$ \mathrm{I}\mathrm{r}{{\left(\mathrm{C}\hat{\mkern6mu} \mathrm{N}\right)}_3}^{+}+\mathrm{I}\mathrm{r}{{\left(\widehat{\mathrm{C}}\mathrm{N}\right)}_3}^{-}\to {}^{3\ast}\mathrm{I}\mathrm{r}{\left(\widehat{\mathrm{C}}\mathrm{N}\right)}_3+\mathrm{I}\mathrm{r}{\left(\widehat{\mathrm{C}}\mathrm{N}\right)}_3 $$

Thus, the excited ^3*^Ir(C^N)_3_ states can be produced via three different routes depending on the values of redox potentials of the given oxidant/reductant pair involved in the overall reaction scheme. Depending on the nature of Ir(C^N)_3_ chelates, some of the possible excitation channels can be more operative due to their more exergonic character. Semiquantitative discussion may be possible taking into account the values of redox potentials characterizing TPrA^+^/TPrA, Ir(C^N)_3_^+^/Ir(C^N)_3_, Ir(C^N)_3_/Ir(C^N)_3_^−^, and TPrA^+^/TPrA^•^ redox couples because these values allow the estimation of the amount of energy released in the given electron transfer process possibly populating the excited ^3*^Ir(C^N)_3_ species. When TPrA^•^ radical is not a strong enough reductant to produce Ir(C^N)_3_^−^ anion, one can simply exclude processes 6f and 6 g from considerations. The difference between values of the redox potentials of Ir(C^N)_3_^+^/Ir(C^N)_3_ and TPrA^+^/TPrA^•^ redox couples seems to be the most crucial parameter. It can be simply argued that the energy released in the electron transfer between Ir(C^N)_3_^+^ oxidant and TPrA^•^ reductant must be sufficient to populate the excited ^3*^Ir(C^N)_3_ state to observe efficient ECL excitation; otherwise process 6f will be not operative. Taking into account that TPrA^•^ radical is a strong reductant with redox potential close to −1.5 V versus normal hydrogen electrode NHE (or ca. −2.05 V versus FC/FC^+^), one can expect that Ir(pq)_3_/TPrA system should produce intense ECL emission because the energy released in the electron transfer annihilation between Ir(pq)_3_^+^ and TPrA^•^ is surely enough to populate the excited ^3*^Ir(pq)_3_ state. The analogous processes involving Ir(ppy)_3_ or Ir(24F_2_ppy)_3_ emitters are distinctly less exergonic and Ir(ppy)_3_/TPrA or Ir(24F_2_ppy)_3_ ECL systems should be correspondingly less efficient compared with Ru(bpy)_3_^2+^/TPrA or Ir(pq)/TPrA pairs. No ECL signal was observed for Ir(ppz)_3_/TPrA or Ir(pmi)_3_/TPrA because the electron transfer annihilations within Ir(ppz)_3_^+^/TPrA^•^ or Ir(pmi)_3_^+^/TPrA^•^ systems are not exergonic enough to populate the excited ^3*^Ir(ppz)_3_ or ^3*^Ir(pmi)_3_ species [66, 68].

## ECL properties of the heteroleptic iridium(III) chelates

ECL properties of the heteroleptic iridium(III) complexes containing two equivalent C^N ligands and an additional ancillary ligand have been studied much more extensively compared with the homoleptic ones. It seems to be reasonable because of the wide-ranging combinations of C^N and L^X ligands that can be relatively easily attached to iridium(III) core. The synthetic strategy, first elucidated by Nonoyama [69], involves the reaction of IrCl_3_ × *n*H_2_O with two equivalents of the cyclometalated C^N ligands (cf. Fig. 1). Subsequent reaction, splitting of the resulting μ-dichloro-bridged [Ir(N^C)_2_μ-Cl]_2_ dimer in presence an ancillary L^X ligand, leads to the heteroleptic products in which the N^C ligands are usually in a *trans*-*N*,*N* configuration [70]. Both synthetic steps occur in relatively mild conditions that allow synthesis with more sensitive functionalities, reducing the formation of side products, and simplifying further purification procedures. The applied ancillary ligand can be neutral or negatively-charged that determines the charge of the resulting heteroleptic complex. Monoanionic ligands such as picolinic acid N^OH or acetylacetone O^OH lead to the neutral Ir(C^N)_2_(N^O) or Ir(C^N)_2_(O^O) compounds [70], whereas neutral α-diimine N^N ligands lead to the cationic Ir(C^N)_2_(N^N)^+^ complexes [44]. Correspondingly dianionic ligands like dithiolates HS^SH, monosulfinates HS^SO_2_H, or disulfinates HO_2_S^SO_2_H lead to the anionic Ir(C^N)_2_(S^S)^−^, Ir(C^N)_2_(S^SO_2_)^−^, or Ir(C^N)_2_(O_2_S^SO_2_)^−^ chelates [71].

Presence of different C^N and X^Y ligands in structures of the heteroleptic iridium(III) complexes allows extreme flexibility in the synthesis of different Ir(C^N)_2_(X^Y) luminophores. Depending on the nature of C^N and X^Y ligands attached to the iridium(III) core, the resulting Ir(C^N)_2_(X^Y) complexes exhibit different photo-physical and electrochemical properties. Particularly, the observed emission colors and the values of oxidation and reduction potentials can be precisely tuned through appropriate selection of the cyclometalating C^N and/or auxiliary X^Y ligands. Differences in the photo-physical and electrochemical properties of the given Ir(C^N)_2_(X^Y) complex should, at least to some extent, affect the observed ECL behavior, but the results from the already published ECL studies indicate that extremely high ECL efficiencies seem to be a common feature of the homoleptic Ir(C^N)_3_ and heteroleptic Ir(C^N)_2_(X^Y) complexes.

**Fig. 6:**
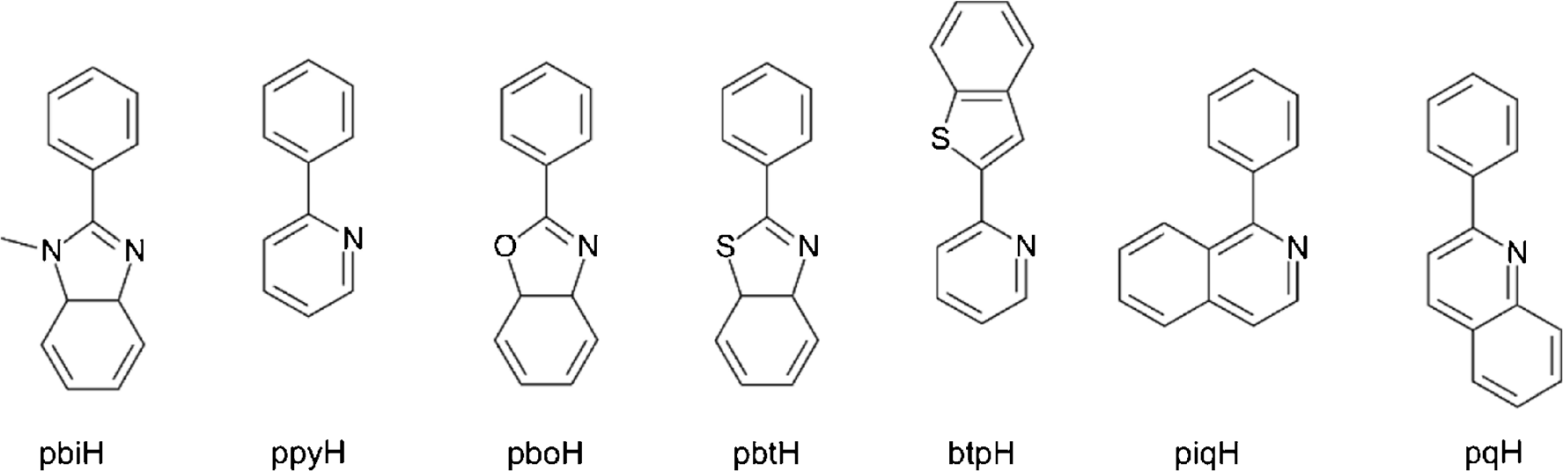
Structures of the protonated forms of C^N ligands used ECL studies of the cyclometalated heteroleptic Ir(C^N)_2_(acac) chelates

Different neutral Ir(C^N)_2_(O^O) or Ir(C^N)_2_(N^O) as well as cationic Ir(C^N)_2_(N^N)^+^ complexes have been successfully tested as ECL luminophores. Among them, particular attention has been paid to Ir(C^N)_2_(O^O) chelates with acetylacetone or other β-diketonate anions and variety of the cyclometalated C^N ligands (structures depicted in Fig. 6). ECL studies of the cyclometalated iridium(III) complexes with the general formula Ir(C^N)_2_(acac) have been presented in reports from the Author laboratory [72–74], describing the generation of the excited ^3*^Ir(C^N)_2_(acac) studied by means of a triple-potential-step technique in 0.1 M (*n*-C_4_H_9_)_4_NPF_6_ acetonitrile/dioxane (1:1) solutions. Electron transfer annihilation between the electrochemically generated Ir(C^N)_2_(acac)^+^ and A^−^ species (radical anions of aromatic nitriles) lead to very efficient generation of ECL emission with extremely high ECL efficiencies (up to 0.40) close to the excited ^3*^Ir(C^N)_2_(acac) luminescence quantum yields. The same ECL excitation mechanism (Equations 7a–7d) was proposed for Ir(C^N)_2_(acac) chelates with different C^N ligands such as 2-phenylpyridine [73], 1-phenyl-*iso*-quinoline [73], 2-(2-pyridyl)benzothiophene [73], 2-phenylbenzothiazole [72], 2-phenylbenzoxazole [73], 2-phenyl-N-methyl-benzimidazole [74], and their substituted derivatives.

7a $$ \mathrm{I}\mathrm{r}{\left(\mathrm{C}\hat{\mkern6mu} \mathrm{N}\right)}_2\left(\mathrm{acac}\right)-{e}^{-}\rightleftarrows \mathrm{I}\mathrm{r}{\left(\mathrm{C}\hat{\mkern6mu} \mathrm{N}\right)}_2{\left(\mathrm{acac}\right)}^{+} $$

7b $$ \mathrm{A}+{e}^{-}\rightleftarrows {\mathrm{A}}^{-} $$

7c $$ \mathrm{I}\mathrm{r}{\left(\mathrm{C}\hat{\mkern6mu} \mathrm{N}\right)}_2{\left(\mathrm{acac}\right)}^{+}+{\mathrm{A}}^{-}\to \mathrm{I}\mathrm{r}{\left(\mathrm{C}\hat{\mkern6mu} \mathrm{N}\right)}_2\left(\mathrm{acac}\right)+\mathrm{A} $$

7d $$ \mathrm{I}\mathrm{r}{\left(\mathrm{C}\hat{\mkern6mu} \mathrm{N}\right)}_2{\left(\mathrm{acac}\right)}^{+}+{\mathrm{A}}^{-}\to {}^{3\ast}\mathrm{I}\mathrm{r}{\left(\widehat{\mathrm{C}}\mathrm{N}\right)}_2\left(\mathrm{acac}\right)+\mathrm{A} $$

When the electron transfer annihilation between Ir(C^N)_2_(acac)^+^ and A^−^ species is sufficiently exergonic, the excited ^3*^Ir(C^N)_2_(acac) state can be nearly quantitatively populated with efficiencies in the range of 60–100 % (cf. data in Table 1). The reported results have unambiguously supported applicability of the cyclometalated iridium(III) chelates in the design of new and very effective ECL systems emitting over the entire range of the UV-VIS radiation (Table 1).

**Table 1:** Summary of the spectroscopic, electrochemical and electrochemiluminescence data for the mixed Ir(C^N)(acac)^+^/A^−^ ECL systems in 0.1 M (*n*-C_4_H_9_)_4_NPF_6_ acetonitrile/dioxane 1:1 solutions

Iridium(III) chelate	${\tilde{\nu}}_{\mathrm{em}}$ /cm^−1^	*ϕ*_em_^a^	*E*_ox_/V	Organic co-reactant	*E*_red_/V	*ΔG*_es_/eV	*ϕ*_ecl_^a^	*ϕ*_es_
Ir(2,4,5F_3_pbi)_2_(acac)^b^	20490	0.12	+0.74	4,4′-dicyano-*p*-biphenyl	−2.12	−0.32	0.12	1.00
Ir(2,4F_2_pbi)_2_(acac)	20300	0.28	+0.59	4,4′-dicyano-*p*-biphenyl	−2.12	−0.19	0.21	0.75
Ir(2,5F_2_pbi)_2_(acac)	19920	0.31	+0.61	4,4′-dicyano-*p*-biphenyl	−2.12	−0.26	0.27	0.87
Ir(3,5F_2_pbi)_2_(acac)	19530	0.48	+0.51	4,4′-dicyano-*p*-biphenyl	−2.12	−0.21	0.46	0.96
Ir(4Fpbi)_2_(acac)	20660	0.27	+0.44	4,4′-dicyano-*p*-biphenyl	−2.12	−0.05	0.20	0.74
Ir(pbi)_2_(acac)	20450	0.47	+0.30	1-cyanonaphthalene	−2.34	−0.22	0.34	0.72
Ir(ppy)_2_(acac)	19010	0.72	+0.40	4,4′-dicyano-*p*-biphenyl	−2.12	−0.16	0.55	0.76
Ir(4Fpbo)_2_(acac)	19380	0.44	+0.76	1,4-dicyanobenzene	−2.03	−0.39	0.25	0.57
Ir(pbo)_2_(acac)	18640	0.38	+0.60	1,4-dicyanobenzene	−2.03	−0.32	0.34	0.89
Ir(4Fpbt)_2_(acac)	18350	0.54	+0.70	4-acetylbenzonitrile	−1.96	−0.39	0.37	0.69
Ir(4CH_3_Opbt)_2_(acac)	18250	0.46	+0.51	1,4-dicyanobenzene	−2.03	−0.28	0.38	0.83
Ir(4CH_3_pbt)_2_(acac)	17850	0.41	+0.53	1,4-dicyanobenzene	−2.03	−0.35	0.22	0.54
Ir(2,3F_2_pbt)_2_(acac)	17790	0.46	+0.72	1,4-dicyanobenzene	−2.03	−0.24	0.35	0.70
Ir(pbt)_2_(acac)	17670	0.44	+0.57	1,4-dicyanobenzene	−2.03	−0.41	0.32	0.71
Ir(4CF_3_pbt)_2_(acac)	17360	0.32	+0.79	1,4-dicyanonaphthalene	−1.73	−0.37	0.29	0.91
Ir(btp)_2_(acac)	16300	0.12	+0.36	1,4-dicyanobenzene	−2.03	−0.37	0.08	0.75
Ir(piq)_2_(acac)	15570	0.47	+0.47	1,4-dicyanobenzene	−2.03	−0.51	0.20	0.65

Luminescence maxima ${\tilde{\nu}}_{\mathrm{em}}$ and luminescence quantum efficiencies *ϕ*_em_, redox potentials for one-electron oxidation of Ir(C^N)_2_(acac) *E*_ox_ versus FC/FC^+^ and one-electron reduction of organic co-reactants A *E*_red_, standard free energies of the excited state population *ΔG*_es_ (as calculated from $\varDelta {G}_{\mathrm{es}}=F\left({E}_{\mathrm{red}}-{E}_{\mathrm{ox}}\right)+hc{\tilde{\nu}}_{\mathrm{em}}$ relationship), ECL efficiencies *ϕ*_ecl_ and efficiencies of the excited ^3*^Ir(C^N)(acac) population *ϕ*_es_. Data taken from refs. [72–74]

^a^ECL efficiency *ϕ*_ecl_ = 0.05 for Ru(bpy)_3_^2+^/Ru(bpy)_3_^+^ pair and quantum yield of emission from the excited ^3*^Ru(bpy)_3_^2+^ *ϕ*_em_ = 0.06 have been found in acetonitrile solutions at room temperature

^b^Abbreviations 2,4,5F_3_pbi, 2,4F_2_pbi, 2,5F_2_pbi, 3,5F_2_pbi, and 4Fpbi are used for substituted 2-(2,4,5-trifluorophenyl)-*N*-methyl-imidazole, 2-(2,4-difluorophenyl)-*N*-methyl-imidazole, 2-(2,5-difluorophenyl)-N-methyl-imidazole, 2-(3,5-difluorophenyl)-*N*-methyl-imidazole, and 2-(4-fluorophenyl)-*N*-methyl-imidazole ligands, respectively. The same abbreviations system is used for pboH and pbtH derivatives. Structures of the unsubstituted pbiH, ppyH, pboH, pbtH, btpH, and piqH ligands are depicted in Fig. 5

Taking into account the data from Tables 1 and 2, one can expect that the electron transfer between the oxidized Ir(C^N)_2_(acac)^+^ species and any strong enough reductant will lead to quite efficient generation of the excited ^3*^Ir(C^N)_2_(acac) states. Particularly the reduced Ir(C^N)_2_(acac)^−^ species or the electrochemically generated TPrA^•^ radical are strong enough reductants that allows observation of very efficient ECL excitation in the way similar to that found for Ir(ppy)_3_ luminophore:

8a $$ \mathrm{I}\mathrm{r}{\left(\mathrm{C}\hat{\mkern6mu} \mathrm{N}\right)}_2{\left(\mathrm{acac}\right)}^{+}+\mathrm{I}\mathrm{r}{\left(\mathrm{C}\hat{\mkern6mu} \mathrm{N}\right)}_2{\left(\mathrm{acac}\right)}^{-}\to {}^{3\ast}\mathrm{I}\mathrm{r}{\left(\widehat{\mathrm{C}}\mathrm{N}\right)}_2\left(\mathrm{acac}\right)+\mathrm{I}\mathrm{r}{\left(\mathrm{C}\hat{\mkern6mu} \mathrm{N}\right)}_2\left(\mathrm{acac}\right) $$

8b $$ \mathrm{I}\mathrm{r}{\left(\mathrm{C}\hat{\mkern6mu} \mathrm{N}\right)}_2{\left(\mathrm{acac}\right)}^{+}+{\left(n\hbox{-} {\mathrm{C}}_3{\mathrm{H}}_7\right)}_2{\mathrm{NC}}^{\bullet }{\mathrm{H}\mathrm{CH}}_2{\mathrm{C}\mathrm{H}}_3\to {}^{3\ast}\mathrm{I}\mathrm{r}{\left(\mathrm{C}\hat{\mkern6mu} \mathrm{N}\right)}_2\left(\mathrm{acac}\right)+{\left(n\hbox{-} {\mathrm{C}}_3{\mathrm{H}}_7\right)}_2{\mathrm{NC}}^{+}{\mathrm{H}\mathrm{CH}}_2{\mathrm{C}\mathrm{H}}_3 $$

**Table 2:** Summary of the spectroscopic, electrochemical and electrochemiluminescence data for the mixed Ir(C^N)(O^O)/TPrA ECL systems in 0.1 M (*n*-C_4_H_9_)_4_NPF_6_ acetonitrile solutions

Iridium(III) chelate	*λ*_em_ /nm	*ϕ*_em_	*E*_ox_/V	*I*_ecl_^a^	Ref
Ir(fpp)_2_(acac)	545	0.23	+0.35	13.5	[77]
Ir(fpbi)_2_(acac)	500	0.15	+0.28	2.5	[77]
Ir(mdx)_2_(acac)	520	0.13	+0.67	11.5	[77]
Ir(pq)_2_(tmd)	590	0.10	+0.49	26.0	[75]
Ir(pq)_2_(dbm)	588	0.03	+0.66	6.5	[76]
Ir(pq)_2_(acac)	589	0.10	+0.57	77.0	[75]
Ir(pq)_2_(acac)	610	0.60	+0.53	10.3	[62]
Ir(35Me_2_pq)(acac)^b^	622	0.42	+0.35	37.6	[62]
Ir(354Me_3_pq)(acac)	608	0.66	+0.34	20.8	[62]
Ir(24F_2_ppy)(avo)	629	0.014	+0.56	2	[78]
Ir(pbt)_2_(avo)	563	0.025	+0.38	11	[78]
Ir(ppy)_2_(avo)	513	0.017	+0.22	0.03	[78]
Ir(ppy)_2_(acac)	519	0.11	+0.26	0.96	[78]
Ir(pbt)_2_(acac)	562	0.22	+0.43	2214	[78]

Luminescence maxima *λ*_em_ and luminescence quantum efficiencies *ϕ*_em_, redox potentials for one-electron oxidation of Ir(C^N)_2_(O^O) *E*_ox_ versus FC/FC^+^ and ECL signal intensities *I*_ecl_. Data taken from refs. [62, 75–78]

^a^ECL intensities *I*_ecl_ as related to Ru(bpy)_3_^2+^/TPrA reference system with the ECL intensity *I*_ecl_(ref) taken as unity.

^b^Abbreviations 3,5Me_2_pq and 354Me_3_pq denote 2-(3,5-dimethylphenyl)-quinoline and 2-(3,5-dimethylphenyl)-4-methylquinoline ligands. Structures of the unsubstituted pbiH, ppyH, pboH, pbtH, btpH, and piqH ligands are depicted in Fig. 5

**Fig. 7:**
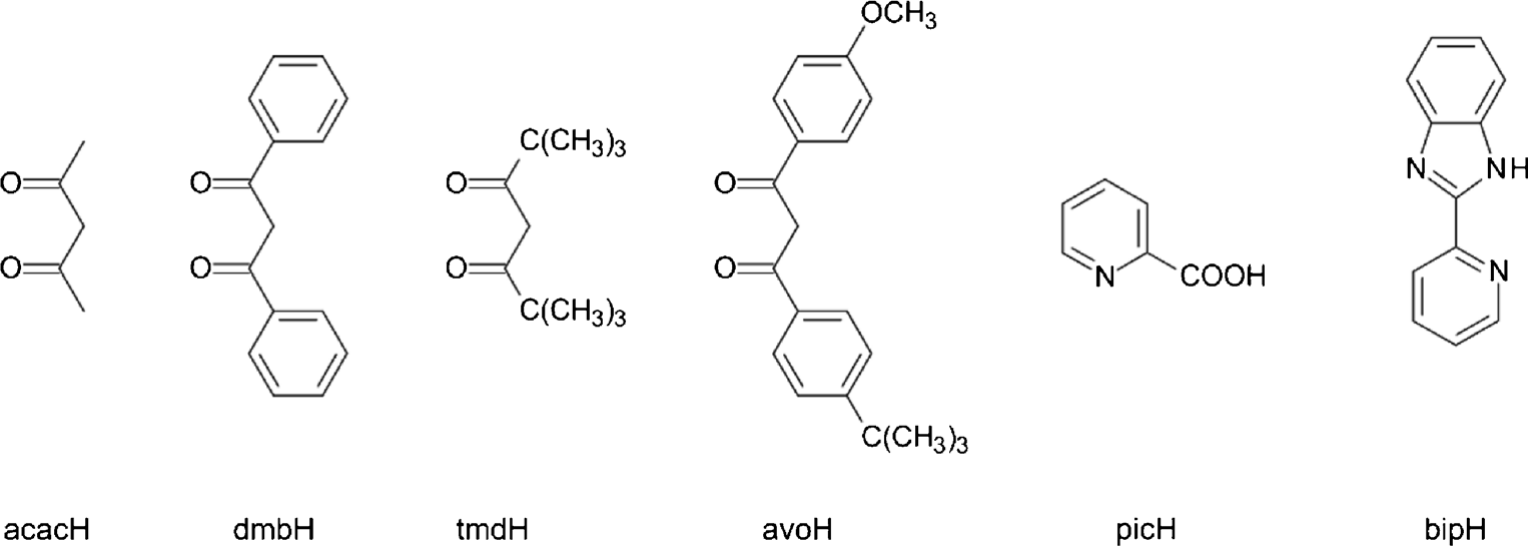
Structures of the protonated forms of O^O, N^O, and N^N ligands used ECL studies of the cyclometalated heteroleptic Ir(C^N)_2_(O^O), Ir(C^N)_2_(N^O), and Ir(C^N)_2_(N^N) chelates

Both types of the ECL excitations, the single and mixed ECL systems, have been investigated. Kim and co-workers [75, 76] have reported the ECL behavior of the red emissive Ir(pq)_2_(O^O) complexes with different monoanionic bidentate ligand such as acetylacetone (acacH), dibenzoylmethane (dbmH) or 2,2′,6,6′-tetramethylhepta-3,5-dione (tmdH) (structures depicted in Fig. 7). Very efficient generation of the excited ^3*^Ir(pq)_2_(acac) or ^3*^(pq)_2_(tmd) states were observed in the annihilation processes as well as in the reaction with TPrA^•^ co-reactant. Additionally to Ir(pq)_2_(acac) and Ir(pq)_3_ chelates, Zhou et al. [62] have investigated Ir(C^N)_2_(acac) derivatives with the methyl substituted 2-phenylquinolines, 2-(3,5-dimethylphenyl)-quinoline − 35Me_2_pqH and 2-(3,5-dimethylphenyl)-4-methylquinoline − 354Me_2_pqH. It should be noted, however, that quite different data (e.g., ECL efficiencies) for ^3*^Ir(pq)_2_(acac)/TPrA system have been presented in References [62] and [75, 76]. Moreover, ECL efficiencies for the single ECL systems, Ir(pq)_2_(acac)^+^/Ir(pq)_2_(acac)^−^ − 0.16 and Ir(pq)_2_(tmd)^+^/Ir(pq)_2_(tmd)^−^ − 0.80, distinctly larger than the emission quantum yields (0.10 for both ^3*^Ir(pq)_2_(acac) and ^3*^Ir(pq)_2_(tmd) emitters) have been reported [76]. With the Ru(bpy)_3_^3+^/Ru(bpy)_3_^+^ reference efficiency equal to 0.05, one can obtain quite unreasonable *ϕ*_ecl_ values of 3.6 or 8.85, distinctly exceeding the maximally possible *ϕ*_ecl_ = 1 limit. Similarly, annihilation ECL efficiencies for Ir(35Me_2_pq)_2_(acac)^+^/Ir(pq)_2_(35Me_2_acac)^−^ or Ir(354Me_3_pq)_2_(acac)^+^/Ir(pq)_2_(354Me_3_acac)^−^ pairs [62], reported to be 72 and 177 times larger compared with Ru(bpy)_3_^3+^/Ru(bpy)_3_^+^ reference system, seem to be somewhat doubtful. Although the heteroleptic cyclometalated iridium(III) chelates, Ir(pq)_2_(acac) and its analogues, can be treated as very promising alternatives to Ru(bpy)_3_^2+^ in any ECL-based analytical application, additional more precise investigations seem to be absolutely necessary for more quantitative confirmation.

Fairly efficient ECL excitation have been reported by Wan et al. [77] for Ir(C^N)_2_(acac)/TPrA^•^ systems based on the green emissive Ir(C^N)_2_(acac) complexes with 2-(4-fluorophenyl)-4-phenylpyridine (fppH), 1-(4-fluorobenzyl)-2-(4-fluorophenyl)-1*H*-benzo[*d*] imidazole (fpbiH), or 2,5-di-*p*-tolyl-1,3,4-oxadiazole (mdxH) cyclometalating ligands C^N. Zhou, Qi et al. [78] have reported ECL studies for the green, yellow, and red emissive Ir(C^N)_2_(O^O) complexes with the cyclometalated 24F_2_ppy, ppy, or pbt C^N ligands and acac or avobenzone (1-(4-methoxyphenyl)-3-(4-*tert*-butylphenyl)propane-1,3-dione − avo) as the ancillary O^O ligands. Noteworthy, 2, 11, and 214 times higher ECL efficiencies than for the Ru(bpy)_3_^2+^/TPrA reference system under identical conditions have been reported for the Ir(24F_2_ppy)_2_(avo)/TPrA, Ir(pbt)_2_(avo)/TPrA, and Ir(pbt)_2_(acac)/TPrA pairs. TPrA co-reactant has been also used to generate ECL emission from the Ir(C^N)_2_(acac) chelate with the photochromic, an open o-dte or closed c-dte form of 1,2-*bis*[2-methyl-5-(2-pyridyl)-3-thienyl]cyclopentene − dteH ligand attached to the central iridium(III) ion. Credi et al. [79] have studied ECL behavior of two Ir(o-dte)_2_(acac) and Ir(c-dte)_2_(acac) photo-isomers observing ECL excitation of Ir(o-dte)_2_(acac) form, whereas no ECL signal was detected for Ir(c-dte)_2_(acac).

ECL behavior of Ir(C^N)_2_(N^O) chelates has been found to be similar to that reported for Ir(C^N)_2_(O^O). Particularly the Ir(C^N)_2_(pic) chelates containing benzimidazole or 2-phenylquinoline derivatives as the cyclometalating C^N ligands and picolinic acid anion − pic as the ancillary N^O ligand exhibit ECL activity comparable to their Ir(C^N)_2_(acac) analogues [74, 76]. ECL studies performed for iridium(III) *bis*(2-(*p*-tolyl)pyridinato-N, C^2^′)(picolinate) − Ir(tpy)_2_(pic) complex have shown that intense green emission from the excited ^3*^Ir(tpy)_2_(pic) state can be observed from all three modes of ECL generation, i.e., Ir(tpy)_2_(pic)^+^ + Ir(tpy)_2_(pic)^−^ annihilation, oxidative-reduction within Ir(tpy)_2_(pic)/TPrA system, and reductive-oxidation within Ir(tpy)_2_(pic)/S_2_O_8_^2−^ system [80]. Mechanism of ECL excitation within Ir(tpy)_2_(pic)/S_2_O_8_^2−^ system has been proposed as

9a $$ {\mathrm{S}}_2{{\mathrm{O}}_8}^{2-}+{e}^{-}\to {{\mathrm{S}\mathrm{O}}_4}^{2-}+{{\mathrm{S}\mathrm{O}}_4}^{\bullet -} $$

9b $$ \mathrm{I}\mathrm{r}{\left(\mathrm{t}\mathrm{p}\mathrm{y}\right)}_2\left(\mathrm{pic}\right)+{e}^{-}\to \mathrm{I}\mathrm{r}{\left(\mathrm{t}\mathrm{p}\mathrm{y}\right)}_2{\left(\mathrm{pic}\right)}^{-} $$

9c $$ \mathrm{I}\mathrm{r}{\left(\mathrm{t}\mathrm{p}\mathrm{y}\right)}_2{\left(\mathrm{pic}\right)}^{-}+{{\mathrm{SO}}_4}^{\bullet -}\to {}^{3\ast}\mathrm{I}\mathrm{r}{\left(\mathrm{t}\mathrm{p}\mathrm{y}\right)}_2\left(\mathrm{pic}\right)+{{\mathrm{SO}}_4}^{2-} $$

Typically, in the reductive-oxidation process with peroxodisulfate S_2_O_8_^2−^ anion, Ir(tpy)_2_(pic)produces 8-fold more efficient ECL signal than Ru(bpy)_3_^2+^ under similar conditions. Noteworthy, the oxidative-reduction process with TPrA lead to much less efficient generation of ^3*^Ir(tpy)_2_(pic) species because the electron transfer from TPrA^•^ to Ir(tpy)_2_(pic)^+^ is thermodynamically insufficient to populate the excited to ^3*^Ir(tpy)_2_(pic) state. Song et al. have investigated ECL properties of Ir(ppy)_2_(N^O) and Ir(pq)_2_(N^O) complexes with the anionic N^O co-ligands, deprotonated forms of *N*-phenylmethacrylamide, *N*-phenylacetamide, *N*-phenylbenzamide, and *N*-naphthylbenzamide [81]. In the reported ECL experiments, TPrA was used as the co-reactant, with the results indicating a critical role of the ancillary ligands in the design and selection of iridium(III) complexes for any practical ECL applications. ECL efficiencies measured for the iridium(III) chelates investigated by Song et al. [81] were reported to be very small, orders of magnitude smaller than characteristic for Ir(C^N)_2_(pic) or Ir(C^N)_2_(acac) chelates. Another class of the negatively charged N‑heterocyclic carbene C^C ancillary phenyl-substituted imidazolylidene ligands has been investigated by Hogan et al. [82]. The reported Ir(ppy)_2_(C^C) complexes were tested for the ECL excitation in the electron transfer annihilation between the reduced Ir(ppy)_2_(C^C)^−^ and oxidized Ir(ppy)_2_(C^C)^+^ forms. Despite the investigated ^3*^Ir(ppy)(C^C) species being nicely emissive with high (0.42–0.68) luminescence quantum yields, the ECL excitation was usually distinctly less efficient compared with the reference Ru(bpy)_3_^3+^/Ru(bpy)_3_^+^ system. Moreover, in most cases, lack of the ECL emission was observed when TPrA was applied as ECL co-reactant, most probably because TPrA^•^ radical is not a strong enough reductant to populate the excited ^3*^Ir(ppy)(C^C) states by means of the electron transfer between ^3*^Ir(ppy)(C^C)^+^ and TPrA^•^ species. Very recently, Qi et al. [83] reported ECL studies of novel Ir(24F_2_ppy)_2_(bip) complex with the negatively charged ancillary N^N ligand – the deprotonated form of 2-(2-pyridyl)benzimidazole − bipH. Intense green ECL emissions, characteristic for the ^3*^Ir(24F_2_ppy)_2_(bip) excited state, were observed in the annihilation and co-reactant processes. In the reported ECL studies, TPrA and benzoyl peroxide − BPO ECL co-reactants were tested. During electrochemical reduction, BPO molecule is decomposed with production of strong oxidant C_6_C_5_CO_2_^•^ radical

10a $$ \mathrm{B}\mathrm{P}\mathrm{O}+{e}^{-}\to {\mathrm{C}}_6{\mathrm{H}}_5{{\mathrm{C}\mathrm{O}}_2}^{-}+{\mathrm{C}}_6{\mathrm{H}}_5{{\mathrm{C}\mathrm{O}}_2}^{\bullet } $$

10b $$ \mathrm{I}\mathrm{r}{\left(24{\mathrm{F}}_2\mathrm{p}\mathrm{p}\mathrm{y}\right)}_2\left(\mathrm{b}\mathrm{i}\mathrm{p}\right)-{e}^{-}\to \mathrm{I}\mathrm{r}{\left(24{\mathrm{F}}_2\mathrm{p}\mathrm{p}\mathrm{y}\right)}_2{\left(\mathrm{b}\mathrm{i}\mathrm{p}\right)}^{-} $$

10c $$ \mathrm{I}\mathrm{r}{\left(24{\mathrm{F}}_2\mathrm{p}\mathrm{p}\mathrm{y}\right)}_2{\left(\mathrm{b}\mathrm{i}\mathrm{p}\right)}^{-}+{\mathrm{C}}_6{\mathrm{H}}_5{{\mathrm{C}\mathrm{O}}_2}^{\bullet}\to {}^{3\ast}\mathrm{I}\mathrm{r}{\left(24{\mathrm{F}}_2\mathrm{p}\mathrm{p}\mathrm{y}\right)}_2\left(\mathrm{b}\mathrm{i}\mathrm{p}\right)+{\mathrm{C}}_6{\mathrm{H}}_5{{\mathrm{C}\mathrm{O}}_2}^{-} $$

The electrochemically generated C_6_C_5_CO_2_^•^ intermediate is a strong enough oxidant to oxidize the reduced Ir(24F_2_ppy)_2_(bip)^−^ species with efficient population of the excited ^3*^Ir(24F_2_ppy)_2_(bip) state.

**Fig. 8:**
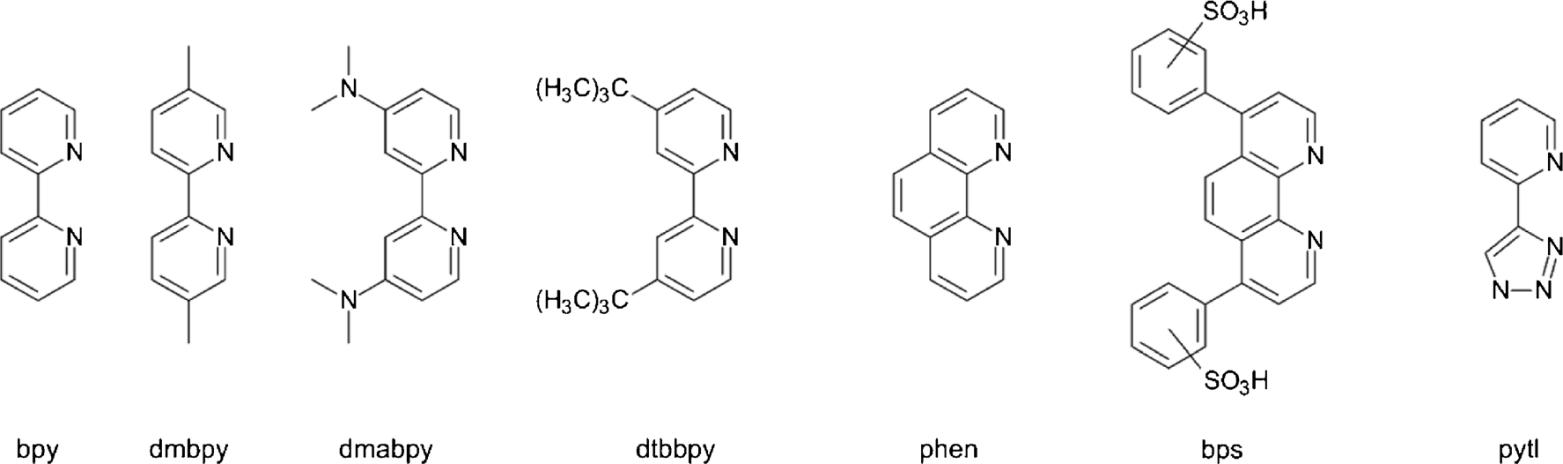
Structures of N^N ligands used ECL studies of the cyclometalated heteroleptic Ir(C^N)_2_(N^N)^+^ chelates

The last of the iridium(III) chelates described above, the Ir(24F_2_ppy)_2_(bip) complex from Ir(C^N)_2_(N^N) family, can be treated as a border case between the neutral Ir(C^N)_2_(X^X) and the positively charged Ir(C^N)_2_(N^N)^+^ complexes with an ancillary α-diimine N^N ligands (structures presented in Fig. 8). Similarly to the neutral Ir(C^N)_2_(X^X) complexes, their Ir(C^N)_2_(N^N)^+^ counterparts are usually strongly emissive and exhibit stable redox characteristics. Moreover, the positively charged Ir(C^N)_2_(N^N)^+^ chelates are distinctly more soluble in the polar solvents, and consequently more suitable for ECL studies in an aqueous media, especially when the N^N ligand attached to the Ir(C^N)_2_^+^ core is further modified to increase solubility. Because solubility of an ECL luminophore in water is crucial for most of the ECL-based analytical applications, Ir(C^N)_2_(N^N)^+^ chelates have been extensively studied beginning from the first communication of Lee et al. [75] presenting ECL behavior of the prototype Ir(ppy)_2_(phen)^+^ and Ir(ppy)_2_(bpy)^+^ complexes with 1,10-phenathroline − phen or 2,2´-bipyridine − bpy N^N co-ligands. Both the ECL Ir(ppy)_2_(phen)^+^/TPrA and Ir(ppy)_2_(bpy)^+^/TPrA systems investigated in acetonitrile solutions were reported to be more efficient than the reference ECL system Ru(bpy)_3_^2+^/TPrA. Similar results have been reported by Francis et al. [84] for Ir(ppy)_2_(bpy)^+^, Ir(ppy)_2_(phen)^+^, and Ir(ppy)_2_(pbs)^−^ chelates applied in the chemiluminescence – CL detection in acidic aqueous solutions. Bathophenanthroline disulfonate ligand − bps (i.e., 4,7-diphenyl-1,10-phenanthrolinedisulfonate dianions) has also been applied as ancillary N^N ligands in Ir(24F_2_ppy)_2_(bps)^−^, Ir(pbt)_2_(bps)^−^, and Ir(piq)_2_(bps)^−^ luminophores [84–86]. These complexes were successfully utilized in the CL reactions with cerium(IV) and organic analytes. Although the cited works from the Francis group are not exactly devoted to the ECL excitation, the presented results are worthy to be mentioned because of the direct analogy between CL and ECL processes. Results presented by Zhou et al. [87] for different Ir(C^N)_2_(bps)^−^/TPrA ECL systems support the latter conclusion, despite the ECL intensities that were found to be lower than that of the Ru(bpy)_3_^2+^/TPrA reference system.

A family of new functional *bis*-cyclometalated thiophene-based cationic iridium complexes has been studied by Marcaccio et al. [88]. The reported Ir(C^N)_2_(N^N)^+^ chelates with the C^N type ligands such as 2-thienylpyridine − tpyH, 5-(2-pyridyl)-2-methylthiophene − 5CH_3_tpyH, and 5-(2-pyridyl)thiophene-2-carbaldehyde − 5CHOtpyH, together with N^N ligands (phen, bpy, and 5,5′-dimethyl-2,2′-dipyridyl − dmbpy) were applied in the annihilation ECL processes involving the electrochemically generated Ir(C^N)_2_(N^N)^2+^ and Ir(C^N)_2_(N^N) species. The observed ECL efficiencies were, however, rather low, ca. one order of magnitude lower than characteristic for the reference Ru(bpy)_3_^3+^/Ru(bpy)_3_^+^ system. It can be tentatively explained by rather low emission quantum yields (0.005–0.018) of the excited ^3*^Ir(C^N)_2_(N^N)^+^ states under study. Somewhat more exciting results have been reported by Mei et al. [89] for the Ir(C^N)_2_(bpy)^+^ chelates containing 2-phenyquinoline derivatives as the cyclometalating C^N ligands. The investigated complexes, with the emission quantum yields between 0.011 and 0.085, were tested as active ECL luminophores in the Ir(C^N)_2_(bpy)^+^/TPrA systems. The observed ECL intensities, measured in CH_3_CN/H_2_O (40:60 v/v) solutions, were up to 3.8 times larger than found for Ru(bpy)_3_^2+^ under the same experimental conditions. ECL studies of the heteroleptic Ir(C^N)_2_(bpy)^+^, Ir(C^N)_2_(dmabpy)^+^, and Ir(C^N)_2_(dtbbpy)^+^ complexes containing ancillary 2,2′-bipyridine, 4,4′-*bis*(dimethylamino)-2,2′-bipyridine or 4,4′-*bis*(*tert*-butyl)-2,2′-bipyridine N^N ligands have been recently reported by the Ding et al. [90–93]. The ECL mechanisms, spectra, and efficiencies via the annihilation and BPO co-reactant paths have been investigated for four Ir(C^N)_2_(N^N)^+^ complexes with the deprotonated forms of 1-*t*-butyl-4-phenyl-1,2,3-triazole (phtlH) or 1-*t*-butyl-4-(2,4-difluorophenyl)-1,2-3-triazole (24F_2_phtlH) C^N ligands and bpy or dtbbpy ancillary N^N ligands [90]. In the case of Ir(C^N)_2_(N^N)^2+^/Ir(C^N)_2_(N^N) annihilation ECL systems, the obtained ECL efficiencies were found to be less compared with the reference Ru(bpy)_3_^2+^/Ru(bpy)^+^ system. Co-reactant Ir(C^N)_2_(N^N)^+^/BPO systems have been found to be up to 3.5-4.5 times more efficient. Similar annihilation ECL behavior has also been found for Ir(ppy)_2_(bpy)^+^, Ir(ppy)_2_(dtbbpy)^+^, and Ir(ppy)_2_(dmabpy)^+^chelates and their analogues with 2-(2,4-*di*-fluorophenyl)-pyridine or 2-(2,4-*di*-fluorophenyl)-5-methyl-pyridine C^N ligands [91, 92]. Among the Ir(C^N)_2_(N^N)^+^ complexes investigated by Ding et al., the most efficient annihilation ECL system based on Ir(ppy)_2_(bpy)^+^ chelate had ECL efficiency approaching 0.045, close to that 0.05 characteristic for Ru(bpy)_3_^2+^. Interesting self-enhanced ECL has been reported for Ir(24F_2_phtl)_2_(dmabpy)^+^ chelate [93]. The effect was tentatively attributed to the presence of (CH_3_)_2_N− groups acting as a self-co-reactant during the electrochemical oxidation of Ir(24F_2_phtl)_2_(dmabpy)^+^ molecule at sufficiently positive potentials.

ECL properties of the cyclometalated iridium(III)-based fluorophores containing two anionic ppy or 24F_2_ppy ligands and the neutral bidentate 2-(1-substituted-1H-1,2,3-triazol-4-yl)pyridine − pytl ligand have been investigated by Zanarini et al. [94]. The investigated complexes exhibit very intense ECL emission in acetonitrile solutions with ECL efficiencies up to 0.41 for Ir(ppy)_2_(CH_3_pytl)^+^ chelate with 2-(1-methyl-1*H*-1,2,3-triazol-4-yl)pyridine as N^N co-ligands. The obtained ECL efficiencies, similar to those reported for the neutral iridium(III) chelates, allow concluding that the cyclometalated cationic complexes can also be applied in the design of new, extremely efficient ECL systems. Thus, further work in this direction seems to be very promising despite low or moderate ECL efficiency typical for most of the already investigated ECL systems based on Ir(C^N)_2_(N^N)^+^ luminophores. It should be noted, however, that the high excitation efficiency in the annihilation ECL processes performed in the aprotic solvents may be not present in the co-reactant ECL systems usually studied in an aqueous media. ECL luminophores investigated by Zanarini et al. [94] exhibit only moderate ECL efficiencies when TBA is applied as ECL co-reactant. Similar observations have also been reported by Doeven et al. for series of Ir(C^N)_2_(N^N)^+^ chelates incorporating phenylpyridine- and triazolylpyridine-based ligands decorated with methylsulfonate or tetraethylene glycol (TEG) groups [95]. Decoration of C^N and N^N ligands with methylsulfonate or tetraethylene glycol groups was applied to create green or blue emissive ECL luminophores for the ECL excitation in an aqueous media. The investigated highly water soluble complexes were capable of generating moderate ECL signals when TPrA was applied as the ECL co-reactant. However, only one from the investigated complexes, namely Ir(24F_2_ppy)_2_(TEG-pytl)^+^ with tetraethylene glycol substituted pytl N^N ligand, was able to give an ECL intensity approaching that of the Ru(bpy)_3_^2+^/TPrA combination.

Moderate or relatively low efficiencies of the ECL excitation in the co-reactant based ECL systems seem to be a general rule for all of the already investigated Ir(C^N)_2_(N^N)^+^ chelates with different cyclometalating and/or ancillary ligands. Results reported for the complexes Ir(pqcm)_2_(N^N)^+^ with the deprotonated 2-phenyl-quinoline-4-carboxylic acid methyl ester acting as C^N ligand and variety of N^N assistant co-ligands [96] support the above conclusion. Ir(Ir(pqcm)_2_(bpy)^+^, the best of the complexes investigated by Qunbo et al. [96], had three times higher ECL intensity than Ru(bpy)_3_^2+^ when the oxalate C_2_O_4_^2−^ ion was applied as the active ECL co-reactant. Others from the investigated Ir(pqcm)_2_(N^N)^+^ chelates, with more extended π-aromatic N^N co-ligands, were distinctly less efficient, most probably due to smaller values of their emission quantum efficiencies as could be concluded taking into account the same sequence of the ECL intensities and the quantum efficiencies of the excited ^3*^Ir(pqcm)_2_(N^N)^+^ states. Low ECL efficiency reported by Qin [97] for the ionic Ir(ppy)_2_(N^N)^+^ chelate with 4′,5′-dimethyldithiotetrathiafulvenyl[4,5-f][1, 10]phenanthroline co-ligand remains in agreement with the results presented by other authors. The ECL peak intensity of the studied complex was half that of Ir(ppy)_2_(phen)^+^ in the ECL processes investigated in CH_2_Cl_2_ solution containing (*n*-C_4_H_9_)_4_NClO_4_ as the supporting electrolyte and TPrA as the ECL co-reactant. ECL investigations of the Ir(C^N)(bpy)^+^ chelates containing the cyclometalated C^N ligands, anions of 6-methyl-2,4-diphenylquinoline, or 6-methyl-2-(4-methoxyphenyl)-4-phenylquinoline have been recently reported by Song et al. [98]. The reported ECL behavior was found to be similar to that for other previously studied Ir(C^N)(bpy)^+^ chelates.

**Fig. 9:**
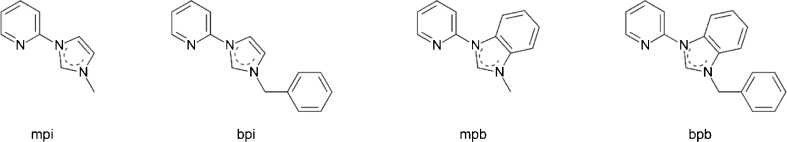
Structures of the N^C ligands used ECL studies of the cyclometalated heteroleptic Ir(C^N)_2_(N^C) chelates

Cyclometalated Ir(C^N)_2_(X^X)^+^ complexes with the ancillary X^X ligands different than more or less modified α-di-imines remain rarely investigated. Only recently Barnard et al. [99] have reported results from the ECL investigations of four cationic heteroleptic iridium(III) complexes prepared using ancillary 3-methyl- or 3-benzyl-substituted, 1-(2-pyridyl)-imidazolylidene − mpi, bpi, or 1-(2-pyridyl)-benzimidazolylidene − mpb, bpb chelating N^C ligands (structures presented in Fig. 9). Owing to rather low (below 0.005) emission quantum yields of the investigated Ir(ppy)_2_(mpi)^+^, Ir(ppy)_2_(bpi)^+^, Ir(ppy)_2_(mpb)^+^, and Ir(ppy)_2_(bpb)^+^ chelates, their applicability in design of any efficient ECL systems could hardly be expected as it was experimentally confirmed in the reported studies of Ir(ppy)_2_(N^C)^+^/TPrA systems based on these chelates.

## ECL analytics with use of the cyclometalated iridium(III) chelates

The above reviewed works have been devoted mainly to the fundamental investigations of ECL processes involving the cyclometalated iridium(III) chelates. Whereas the above statement is exactly true for the annihilation electron transfer excitation, the studies of the co-reactant-based ECL systems were more practically oriented. Particularly the investigations describing the role of TPrA as an active co-reactant seem to be important because attaching the sensitive recognition groups to an iridium(III) ECL active kernel may result in a wide range of specific analytical tools based on ECL emission in similar way as takes place in the case of Ru(bpy)_3_^2+^. An appropriate iridium(III) chelate can be served as a label ECL-based assays applied in the measurement and detection of the given analyte. At the present stage of investigations, however, this possibility seems to be only a very promising option. On the other hand, the recently published works, devoted to different analytical application based on the ECL processes involving iridium(III) chelates, allow the assumption of further intrinsic progress in the field.

During the last few years, different authors have tested the application of different iridium(III) chelates in the detection or determination of different analytes. Song et al. [100] have reported ECL studies of four iridium complexes, in the presence of ammonia as the co-reactant in *N*,*N*-dimethylformamide solution. Among the studied Ir(ppy)_3_, Ir(ppy)_2_(acetylaniline), Ir(ppy)_2_(N-phenylmethacrylamide), and Ir(pq)_2_(acac) complexes, the latter was found to exhibit the highest ECL efficiency with NH_3_. The detection mechanism is based on the electrochemical oxidation of NH_3_ molecule. The deprotonation of the oxidized NH_3_^+^ cation leads to NH_2_^•^ radical, reductant strong enough to produce the excited ^3*^Ir(pq)_2_(acac) state through the electron transfer between NH_2_^•^ and Ir(pq)_2_(acac)^+^ species. At the optimized conditions, linear relationship between the ECL intensity and the NH_3_ concentration was obtain in the concentration range from 1.0 × 10^−7^ to 1.0 × 10^−3^ M with the detection limit of 4 × 10^−8^ M (S/N = 3). The developed method can be applied to the detection of NH_3_ in the atmosphere with recovery close to 100 %. The water-soluble cationic Ir(dpci)_2_(bvbbi)^+^ complex (dpciH = 3,4-diphenylcinnoline; bvbbi = N,N′-bivinylester-1H,1´H-[2,2′] bibenzimidazole) described by Wu et al. [101] has been used for determination of the sulphite SO_3_^2−^ ions. Under optimal experimental conditions, the increased CL response was linear with the concentration of sulphite over the range of 5.0 × 10^−7^–5.0 × 10^−4^ M with the detection limit of the method equal to 1.6 × 10^−7^ M. Although the active reactants were produced using Ce(SO_4_)_2_ × 4H_2_O oxidant, one can expect, according to the analogy between CL and ECL processes, that the applied method can be adapted for ECL-based detection. ECL of Ir(pq)_2_(acac) chelate induced by hydroxide and ethoxide ions have been investigated by Song et al. [102]. The investigated ECL processes involved the studied complex and water in frequently used organic solvents. Hydroxide ions, formed from the dissociation of water traces and Ir(pq)_2_(acac) were electrochemically oxidized to HO^•^ radical and Ir(pq)_2_(acac)^+^ cation, respectively. The HO^•^ could be also produced via a catalytic route by the chemical oxidation HO^−^ + Ir(pq)_2_(acac)^+^ → HO^•^ + Ir(pq)_2_(acac). Subsequently, the excited state ^3*^Ir(pq)_2_(acac) was generated by means of the electron transfer reaction between Ir(pq)_2_(acac)^+^ and HO^•^ species. The emitted light intensity was found to be proportional to the water contents; hence, the method can be used for the determination of water traces in organic solvents. For example, the linear response for amount of water in the range of 0.01–1 % was found for *N*,*N*-dimethylformamide. Similar ranges were also found for dichloromethane, acetonitrile, and acetone. The developed analytical procedure can be used as a possible replacement of the Carl-Fisher titration. Strong ECL emission from ^3*^Ir(pq)_2_(acac) was observed in the presence of the base in the ECL experiment carried out in anhydrous ethanol, indicating that ethoxide ion C_2_H_5_O^−^ (or more specifically ethoxide radical C_2_H_5_O^•^) can also act as an active ECL co-reactant. Molecular chemosensor based on the ECL response to acetate CH_3_CO_2_^−^ anion has been investigated by Schmittel et al. [103]. When structure of Ir(ppy)_2_(phen)^+^ complex is modified by the imidazolium unit decoration of the N^N ligands, the obtained receptor allows quantitative sensing of different anions. Owing to the specific interactions between acetate anion and tri-cationic cavity formed by the positively charged imidazolium units and the iridium center of Ir(ppy)_2_(phen)^+^ core, the investigated ECL luminophore exhibits intrinsic changes of the ECL signature and highly selective ECL enhancement in the presence of CH_3_CO_2_^−^ anions. In a similar way, selective cation sensing becomes possible when Ir(ppy)_2_(phen)^+^ chelate is decorated by attaching the aza-crown macrocycles to the N^N ancillary ligand. The aza-crown ether appended iridium(III) complexes can be applied as ECL probes for metal cations operating on the oxidative-reduction ECL processes with TPrA as co-reactant, as has been presented by Schmittel et al. [104]. Notable sensing of Ag^+^ and Ba^2+^ cations by means of the ECL emission intensity enhancement associated with the bathochromic emission shift was found in contrast to the structurally analogous aza-crown ether appended Ru(phen)_3_^2+^ complexes.

The CL or ECL processes involving the cyclometalated iridium(III) complexes have been successfully applied in the detection of different organic analytes. Zhang and co-workers [105] have described application of the water-soluble cationic Ir(dpci)_2_(bvbbi)^+^ complex in the determination of tryptophan. The applied methodology was based on the inhibition effect of the tryptophan to the CL emission of the previously described Ir(dpci)_2_(bvbbi)^+^/Ce^4+^/SO_3_^2−^ system [101]. Under optimum conditions, the decreased CL intensity was proportional with the concentration of tryptophan in the range from 5.0 × 10^−7^ to 2.0 × 10^−5^ M with the detection limit of 8.2 × 10^−8^ M. The proposed method can be applied for the determination of tryptophan in its pharmaceutical formulations. A similar approach has been applied by Dong et al. in the detection of cysteine [106]. The homoleptic iridium(III) complex with three cyclometalating 1-(2,6-dimethylphenoxy)-4-(4-chlorophenyl)phthalazine ligands were adopted as active luminophore in the presence of potassium permanganate (oxidant) and oxalic acid (co-reactant). Cysteine, exhibiting a sufficient enhancing effect on the intensity of CL emission, could be determined in the linear range from 1.0 × 10^−9^ to 5.0 × 10^−6^ M with the detection limit of 6.9 × 10^−10^ M. Cysteine added in the investigated CL system acts as catalyst allowing more efficient generation of the strong reducing agent CO_2_^−^ through reaction of the cysteine radical with the intermediate HCO_2_^–^ produced during oxidation of oxalic acid. Both reacting intermediates were produced from cysteine and oxalic acid oxidized by potassium permanganate. The analogy between CL and ECL processes allows the expectation that electrochemical oxidation, instead of the use of ordinary Ce^4+^ or MnO_4_^−^ oxidants, can be applied to produce analytically useful signal. Li and co-workers [107] described the ECL-based determination of antibiotics (erythromycin or ampicillin) that supports the latter conclusion. The water soluble complex, Ir(pq)_2_(bpy-sugar)^+^ with the N^N ancillary bpy ligand decorated with two 2-hydroxymethyl-tetrahydro-pyran-3,4,5-triol molecules linked via −CH_2_−S− bridges was found to give an ECL signal much higher than that of Ru(bpy)_3_^2+^. The reported results, linear response over the concentration range from 1 nM to 0.5 μM for erythromycin and from 3 nM to 1 μM for ampicillin with the respective detection limits of 0.2 nM and 1 nM, point to good sensitivity and reproducibility of the developed ECL-based analytical protocols. The ECL sensitivity of the investigated iridium(III) chelate to TPrA, much higher than that characteristic for Ru(bpy)_3_^2+^ ion, supports additionally huge analytical potential of the ECL systems based on the cyclometalated iridium(III) chelates. A similar conclusion can be drawn taking into account reported application of Ir(pq)_2_(acac) chelate for use in the flow injection analysis [108]. An aqueous solution containing analyte and Ir(pq)_2_(acac) chelate was passed through the reaction/observation cell with ECL signal generation by means of parallel electrochemical oxidation of the analyte and iridium(III) chelate. In addition to TPrA, different analytes, including oxalate ions, amino acids, aliphatic amines, and NADH, were investigated with findings of particular sensitivity for oxalate ions, tartaric acid, or proline.

## ECL sensing with immobilized cyclometalated iridium(III) chelates

The above-cited examples of analytical applications of the cyclometalated iridium(III) chelates belong to the solution-phase ECL detection with rather limited applications. One of the most significant disadvantages is the amount of the consumed solution containing rather expensive iridium(III) chelate as an ECL probe. Immobilization of the ECL probe on an electrode allows overcoming this limitation, bringing some additional advantages as well. Simple experimental design, possible enhancement of the ECL sensitivity, and the spatial control over the ECL reactions should be mentioned to list some of these advantages. The literature describing more or less advanced immobilization techniques applied for the cyclometalated iridium(III) chelates allows expecting further development of different ECL probes or sensors based on these ECL luminophores.

Similarly as was used in the case of Ru(bpy)_3_^2+^ chelate, several methods allowing the immobilization of the cyclometalated iridium(III) chelates have been tested. The simplest one involves incorporation of the given ECl luminophore into polymer matrices forming film on the electrode surface. This option was tested by Muegge and Richter [109], Tong et al. [110], and Li et al. [111]. Muegge and Richter [109] have investigated three iridium complexes, Ir(24F_2_ppy)(pic), Ir(ppy)_3_, and Ir(pbt)_2_(acac), the emission maxima of which fall in different blue, green, and orange region of the UV-VIS radiation. The investigated complexes were bound in either Nafion or poly(9-vinylcarbazole) matrices, and their ECL behavior was tested with the oxidative-reductive co-reactant TPrA. All three complexes displayed the ECL emission with TPrA when bound in poly(9-vinylcarbazole), whereas only Ir(24F_2_ppy)(pic) was ECL active in the Nafion matrices. The observed ECL signal was obtained from the produced excited ^3*^MLCT states, similarly as it was in the case of the solution-phase excitation. Cationic Ir(pqcm)_2_(bpy)^+^ complex with the cyclometalated ligand, 2-phenyl-quinoline-4-carboxylic acid methyl ester, was investigated by Tong and co-workers [110] in a neutral aqueous solution through immobilization of the iridium complex on a glassy carbon electrode surface with the help of the Nafion film. ECL emission was observed from the electrode immersed in the aqueous solutions containing oxalate ions. The presented ECL sensor gave a linear response for the oxalate concentration from 1.0 × 10^−6^ to 1.0 × 10^−4^ M with the detection limit (S/N = 3) of 9.1 × 10^−7^ M. Two water-insoluble iridium(III) complexes, Ir(ppy)_2_(acac) and Ir(pq)_2_(acac), immobilized in the carbon nanotubes – CNT and Nafion composite films, have been investigated at a glassy carbon electrode by Li et al. [111]. ECL emission from these films was observed in presence of DBAE acting as the ECL co-reactant. The ECL sensors, based on the Ir(ppy)_2_(acac)/Nafion/CNT or Ir(pq)_2_(acac)/Nafion/CNT composite films, were shown to be suitable for the determination of water-soluble substances. For example, the Ir(pq)_2_(acac)/DBAE combination was used to detect codeine with the detection limit of 0.05 μM (S/N = 3). Enhanced ECL emission from ^3*^Ir(ppy)_3_ chelate has been reported by Richter et al. [112] in the presence of hydrophilic ionic liquids − HIL in an aqueous environment. The effect is based on the solid-state sorption of HIL on the electrode surface allowing for increased luminophore and/or TPrA localization and their interaction near the electrode surface. The ability of this adsorption layer to incorporate, extract, or pre-concentrate ECL reagents play a crucial role in the observed phenomenon, formally similar to the incorporation of an ECL luminophore into polymer matrices. ECL properties of Ir(ppy)_2_(bpy)^+^ chelate in block polymer (obtained by means of the ring-opening metathesis polymerization) have been recently presented by Mauzeroll et al. [113].

A much more sophisticated approach, specifically devoted to the ECL-based analytics, involves the use of nanoparticles or nanotubes for immobilization of the cyclometalated iridium(III) complexes. Zanarini et al. [114] reported ECL behavior of the silica nanoparticles loaded with Ir(piq)_2_(acac) chelate. Stable ECL emissions were observed in the aqueous solutions from a completely insoluble neutral Ir(III) complex located inside the silica nanoparticles covered with polyethylene glycol stabilizing shell. The observed ECL emissions were generated using DBAE as the oxidative ECL co-reactant. Immobilization of a water-insoluble iridium complex with organosilica nanoparticles for ECL sensing has also been reported by Liu and Song [115]. Three different kinds of the Ir(pq)_2_(acac) loaded organosilica nanoparticles were prepared using vinyltrimethoxysilane, vinyltriethoxysilane, and phenyltrimethoxysilane precursors, respectively. The prepared nanoparticles were further applied to modify glassy carbon electrodes. The obtained modified electrodes were found to be ECL-active in the solutions containing DBAE. The electrode prepared with the use of the nanoparticles prepared from phenyltrimethoxysilane was found to exhibit the best sensitivity for DBAE with the detection limit of 5.0 × 10^−9^ M and a linear response to DBAE concentration in the range from 1.0 × 10^−8^ to 1.0 × 10^−6^ M. The solid-state ECL behavior of Ir(pq)_2_(*N*-phenylmethacrylamide) has been investigated by Son et al. [116] for the complex immobilized using the multi-wall carbon nanotubes − MWCN. The glassy carbon electrode, coated with the MWCN/Ir(pq)_2_(*N*-phenylmethacrylamide) composite, was used to obtain a well reproducible ECL signal in the presence of TPrA co-reactant.

Immobilization of the cyclometalated iridium(III) luminophores, based on the nanoparticles encapsulation strategy, could allow the fabrication of still more sophisticated ECL sensors. The fabricated sensors can be constructed with the use of water insoluble ECL luminophores that extend their use in aqueous media. Moreover, due to increased sensitivity and specificity of such sensors to different analytes, their quantitative low-level differentiation and/or determination (especially important in the case of bioactive species) may be achieved. Recently published works indicate clearly that this is more than a very promising opportunity. Ultrasensitive ECL technique has been presented by Zhang et al. [117] in the work describing a novel ECL cytosensor for selective evaluation of cell-surface *N*-glycan expression at the single-cell level. Ir(ppy)_2_(dcbpy)^+^ chelate, containing 4,4′-dicarboxy-2,2′-bipyridyl as the ancillary N^N ligand, has been applied for functionalization of the mesoporous silica nanoparticles – MSN. After loading the pores of the MSN scaffolding with Ir(ppy)_2_(dcbpy)^+^ chelate, (3-mercaptopropyl)trimethoxysilane was applied to obtain thiol-modified MSN. Thereafter, Au nanoparticles were assembled onto the MSN colloidal surface. The obtained accumulations were then converted to concanavalin A nanoprobes, used further to modify the glassy carbon electrode coated with graphene functionalized with poly(diallyldimethylammonium chloride). The resulting sandwich-type electrode was used for detection of K562 cells at concentrations ranging from 1.0 × 10^2^ to 1.0 × 10^6^ cells/mL. Lin et al. [118] applied the previously described [107] Ir(pq)_2_(bpy-sugar)^+^ chelate to develop ECL biosensor for the human thrombin. The investigated complex was conjugated through succinimide coupling to the 3-NH_2_-modified thrombin aptamer, and the obtained conjugate was further adsorbed on the magneto-controlled magnetic graphene oxide. In the presence of the target analyte, the aptamers release into solution resulting in increased ECL intensity. The observed ECL intensity was found to have a direct relationship with the thrombin concentration in the range from 2.0 × 10^−9^ to 5.0 × 10^−8^ M with the detection limit of 1.3 × 10^−9^ M (S/N = 3). Song et al. [119] have designed ECL aptasensors for the sensitive detection of fumosin B_1_ using Ir(ppy)_2_(dcbpy)^+^ chelate. The complex was first reacted with mercaptoethylamine to convert the carboxyl − COOH groups of dcbpy ligand into − CONHC_2_H_4_SH. The resulting mercaptoethylamine-Ir complex was used to modify the surface of Au nanoparticles through the Au − S covalent bonds. Further alteration of the modified Au nanoparticles with the aptamer of fumosin B_1_ resulted in construction of ECL-aptasensor. It was accomplished by attaching the modified Au nanoparticles on the surface of an Au electrode covered with the DNA strands partially complementary with fumosin B_1_. ECl signal from the electrode was produced during the positive cyclic voltammetry scan in the solutions containing DBAE as the ECL co-reactant. DNA and fumosin B_1_ competition to the aptamers modified Au nanoparticles leads to ECL signal inversely proportional to the fumosin B_1_ concentration. Standard curve between the fumosin B_1_ concentrations in the range from 0.5 to 50 ng/mL and the ECL intensities from the Au nanoparticles modified with the investigated iridium(III) complex was established. This allowed concluding that the designed ECL aptasensors can be applied in the sensitive detection of mycotoxin.

## Multicolored ECL emission of the cyclometalated iridium(III) chelates

Nearly all of the already published works describing the ECL behavior of the cyclometalated iridium(III) chelates were devoted to the electron transfer excitation of the individual ECL luminophore. Because the same emission wavelength independent ECL excitation mechanism seems to be characteristic for the ECL processes involving the cyclometalated iridium(III) chelates, one could expected parallel excitation of different emissive species when two or more ECL active species are present in the investigated ECL system. This unique opportunity, already postulated and tested by Bruce and Richter [50] at the beginning of ECL studies concerning the cyclometalated iridium(III) chelates, has been recently investigated extensively by Francis et al. [120–122].

Selective ECL excitation of the concomitant Ru(bpy)_3_^2+^ and Ir(24F_2_ppy)(bps)^−^ complexes with TPrA co-reactant in water/acetonitrile (1:1 v/v) solutions have been found to be controlled through the applied electrode potential. Changes in the color of the emitted light from red to green (characteristic for the excited ^3*^Ru(bpy)_3_^2+^ and ^3*^Ir(24F_2_ppy)(bps)^−^ states, respectively), with increasing electrode potential have been explored [120]. Control of the ECL excitation through choice of co-reactant was tested for the Ru(bpy)_2_^3+^ (red emitter) and Ir(ppy)_3_ (green emitter) pair with findings that closely related tertiary-amine ECL co-reactants generate remarkably different emission profiles [121]. Depending on the nature of the applied ECL co-reactant, distinct red or green emission could be observed for some, whereas the others generated both emissions simultaneously. The phenomenon can be rationalized through the relative exergonicities of the electron-transfer processes involving the excited ^3*^Ru(bpy)_2_^3+^ and ^3*^Ir(ppy)_3_ states. Selective excitation of multiple, e.g., red emissive Ru(bpy)_2_^3+^, green emissive Ir(ppy)_3_, and blue emissive Ir(24F_2_ppy)_3_ or Ir(24F_2_ppy)_2_(pic), luminophores has been observed in acetonitrile solutions containing TPrA as ECL co-reactant [122]. Depending on the applied electrode potentials, a quite different emission color was detected. Changes of the observed ECL emission color from green to blue were presented for the ECL system based on the mixture of Ir(ppy)_3_ and Ir(24F_2_ppy)_3_ complexes, whereas the mixture of iridium(III) and ruthenium(II) chelates was able to produce red, yellow, and/or green emissions. The observed emission color for the given combination of the applied ECL luminophores can be tuned changing the used ECL co-reactant as well as the electrode potentials applied for the ECL excitation. As could be expected, the overall reaction mechanisms in the case of multiple ECL emissions are much more complex compared with the simple ECL systems based on the individual ECL luminophore. Not only the nature of the applied ECL chelates and/or ECL co-reactants but also their relative concentrations are responsible for the final emission color and intensity. For more details, the reader is referred to the original papers.

Multicolor ECL emission can be observed not only for ECL processes involving mixtures of the individual ECL luminophores but also for the metal complexes with multiple metal centers localized within a single molecule, as has been presented by Schmittel et al. [123] for di-nuclear Ir^III^ − Ru^II^ and tri-nuclear Ir^III^ − Ru^II^ − Ir^III^ species. Multinuclear chelates studied by Schmittel et al. were constructed from Ru(2,2′:6′,2′′-tertpyridine)_2_^2+^ − Ru(terpy)_2_^2+^ and Ir(pq)_2_(phen)^+^ subunits linked together using − C ≡ C− bridging groups. Co-reactant ECL processes of these species were studied in acetonitrile solutions in the presence of TPrA. Depending on the electrode potential applied for ECL generation different ECL emission band profiles were recorded. The observed changes were rationalized taking into account two possible electron transfer processes involving the oxidized Ru(bpy)_3_^3+^ and Ir(pq)_2_(phen)^2+^ subunits from the parent Ir^III^ − Ru^II^ or Ir^III^ − Ru^II^ − Ir^III^ multinuclear chelates, respectively. Electrochemical oxidation of these species corresponds to the removal of the first electron from the iridium-centered orbitals, whereas the oxidation of the ruthenium terpyridine units is only possible at slightly more positive potentials. Thus, relative contributions of the oxidized Ir^IV^ and Ru^III^ subunits depend on the applied electrode potential. Presence of the spatially separated two different chromophores in the investigated di-nuclear and tri-nuclear complexes allows specific generation of the excited ^3*^Ru(terpy)_2_^2+^ and ^3*^Ir(pq)_2_(phen)^+^ states by means of the electron transfer reaction between Ir^IV^ or Ru^III^ centers and TPrA^•^ radical.

ECL studies of the soft salt containing the complementary charged, cationic Ru(dtbbpy)_3_^2+^ and anionic Ir(ppy)_2_(CN)_2_^−^ complexes in 2:1 ratio have recently been reported by Zysman-Colman et al. [124]. Both annihilation and co-reactant ECL processes involving the investigated salt were explored in acetonitrile solutions with (*n*-C_4_H_9_)_4_NPF_6_ as supporting electrolyte. In the both cases, only emission from the excited ^3*^Ru(dtbbpy)_3_^2+^ state was observed, indicating that the electron transfer excitations leading to the excited ^3*^Ir(ppy)_2_(CN)_2_^−^ state are not operative because of insufficient exergonicities or, optionally, the presence of very efficient quenching processes, e.g., the energy transfer from ^3*^Ir(ppy)_2_(CN)_2_^−^ to Ru(dtbbpy)_3_^2+^. Similar results, excitation of the individual ECL chromophore, have also been reported for the mixture of Ru(bpy)_3_^2+^ and Ir(24F_2_ppy)_2_(bpy)^+^ chelates incorporated into the ion gel comprising triblock polystyrene-*block*-poly(methyl methacrylate)-*block*-polystyrene copolymers and 1-ethyl-3-methylimidazolium *bis*(trifluoromethylsulfonyl)imide ionic liquid. Frisbie at al. [125] reported observation of the ECL emission from the excited ^3*^Ru(bpy)_3_^2+^ (at 610 nm) or ^3*^Ir(24F_2_ppy)_2_(bpy)^+^ (at 540 nm) species for the investigated ECL gel containing only one of the chelate under study. When a blended luminophore system containing a mixture of both chelates was used in the emissive layer, only red-orange-colored emission was observed. Because both of the ECL luminophores could be effectively excited under the applied experimental conditions, lack of green-colored emission in the mixed Ru(bpy)_3_^2+^/Ir(24F_2_ppy)_2_(bpy)^+^ system can be attributed directly to the presence of a very efficient energy transfer process involving ^3*^Ir(24F_2_ppy)_2_(bpy)^+^ and Ru(bpy)_3_^2+^ chelates.

Results presented by Zysman-Colman et al. or Frisbie at al. also allow concluding that design of any new efficient multicolor ECL systems may be a rather difficult task because independent and unperturbed ECL excitation of two or more ECL luminophores comprising the given ECL system can take place only under specific conditions. In the case when the oxidized and reduced forms of two different ECL luminophores annihilate, one can expect the population of different excited states with appropriate partitioning of the electrochemical excitation energy between them. As has been shown by Mussel and Nocera [126], such partitioning strongly depends on energies of the populated excited states. Based on the Marcus theory, one can expect that the ECL luminophore with lower energy of the excited state will be preferentially populated. Efficient population of the excited states for both ECL luminophores involved in the ECL excitation is expected only when their energies are similar, one to another. Real multicolor ECL emission should be more easily realized when the excited states of the applied ECL luminophores are populated through independent excitation channels. It may be more directly realized in the ECL co-reactant systems involving the oxidized or reduced forms of the applied ECL luminophores. In such cases, their excited state should be populated more or less independently, which would allow observation of multiple emissions. This option can be actually realized, as it has been shown by Bruce and Richter or Hogan, Francis et al.

## Concluding remarks

Despite all similarities between the ECL systems based on iridium(III) and ruthenium(II) chelates, there are some distinct differences between them. Whereas ruthenium(II) complexes are usually emissive in the orange/red part of the UV-VIS radiation, their iridium(III) counterparts allow, in good agreement with name of this element, design of new ECL systems with emission over the entire range of rainbow colors, spanning from the near UV to the near IR. This unique possibility arises from the specific photo-physical properties of the cyclometalated iridium(III) chelates that can be precisely tuned through appropriate changes in the ligands environment around the central iridium(III) core. Moreover, emission quantum yields of the excited molecules are usually the case of the cyclometalated iridium(III) chelates, distinctly larger compared with that characterizing the excited ^3*^Ru(bpy)_3_^2+^ ion. Other important ECL advantages of the cyclometalated iridium(III) chelates are well pronounced photochemical and chemical stability together with stability of their oxidized and/or reduced forms. Combination of all of them makes the cyclometalated iridium(III) chelates particularly suitable for designing extremely efficient ECL systems as it has been already realized for the annihilation as well as co-reactant ECL excitation.

Continuous progress in ECL investigations of the cyclometalated iridium(III) chelates has already resulted in good understanding of the phenomena associated with the ECL excitation of these species. Different cyclometalated iridium(III) chelates can be electrochemically excited according to the common mechanism that makes this class of the organometallic luminophores especially suitable for comparative, fundamental, as well as the application oriented ECL studies. The results already reported allow anticipating further development with new insight into the ECL-based analytical applications with distinct improvement of their sensitivity and specificity. Further progress in the design of new, stable, and reproducible ECL probes and sensors utilizing the cyclometalated iridium(III) chelates seems to be obvious. One can also believe that further investigations will bring new interesting results in both liquid and solid media.

Taking into account the current (only ca. 12-y-old) progress in ECL studies of the cyclometalated iridium(III) chelates, one can regard two directions of the further investigations as especially promising. Because efficient parallel ECL excitation has been observed for the ECL systems comprising different ECL luminophores, one can expect the use of the cyclometalated iridium(III) chelates as potential candidates for design of new ECL systems for parallel multi-analyte detection. When two (or more) ECL luminophores would be simultaneously excited with the ECL efficiencies depending on the nature of the given analyte, one could use the emission band shape and the overall emission intensity for qualitative and quantitative analyses, respectively. Immobilization of the title complexes seems to have a brilliant future because it allows bypassing some disadvantages (nobody is perfect) of the cyclometalated iridium(III) chelates. Whereas usual low solubility of these chelates in an aqueous media can be quite easily overcome by appropriate modification of the ligand(s) attached to the central iridium(III) core, quenching processes of their excited states occurring in the presence of the dissolved oxygen are a much more significant problem. To some extent, the quenching processes can be ruled out by means of the nanoparticles encapsulation strategy. ECL systems based on the immobilization of two (or more) ECL luminophores can be particularly interesting.

In summary, the cyclometalated iridium(III) chelates are, without any doubt, worthy of further ECL investigations.
